# A novel ADP-directed chaperone function facilitates the ATP-driven motor activity of SARS-CoV helicase

**DOI:** 10.1093/nar/gkaf034

**Published:** 2025-01-29

**Authors:** Jeongmin Yu, Hyeryeon Im, HyeokJin Cho, Yongmoon Jeon, Jong-Bong Lee, Gwangrog Lee

**Affiliations:** Single-Molecule and Cell Mechanobiology Laboratory, Daejeon, 34141, South Korea; Department of Biological Sciences, Korea Advanced Institute of Science and Technology, Daejeon, 34141, South Korea; Single-Molecule and Cell Mechanobiology Laboratory, Daejeon, 34141, South Korea; Department of Biological Sciences, Korea Advanced Institute of Science and Technology, Daejeon, 34141, South Korea; Single-Molecule and Cell Mechanobiology Laboratory, Daejeon, 34141, South Korea; School of Life Sciences, Gwangju Institute of Science and Technology, Gwangju, 61005, South Korea; Department of Physics, Pohang University of Science and Technology, Pohang, 37673, South Korea; Department of Physics, Pohang University of Science and Technology, Pohang, 37673, South Korea; Single-Molecule and Cell Mechanobiology Laboratory, Daejeon, 34141, South Korea; Department of Biological Sciences, Korea Advanced Institute of Science and Technology, Daejeon, 34141, South Korea

## Abstract

Helicase is a nucleic acid motor that catalyses the unwinding of double-stranded (ds) RNA and DNA via ATP hydrolysis. Helicases can act either as a nucleic acid motor that unwinds its ds substrates or as a chaperone that alters the stability of its substrates, but the two activities have not yet been reported to act simultaneously. Here, we used single-molecule techniques to unravel the synergistic coordination of helicase and chaperone activities, and found that the severe acute respiratory syndrome coronavirus helicase (nsp13) is capable of two modes of action: (i) binding of nsp13 in tandem with the fork junction of the substrate mechanically unwinds the substrate by an ATP-driven synchronous power stroke; and (ii) free nsp13, which is not bound to the substrate but complexed with ADP in solution, destabilizes the substrate through collisions between transient binding and unbinding events with unprecedented melting capability. Our findings provide new insights into how the same enzyme works via two modes on different parts of the substrate and synergistically catalyses the unwinding reaction, utilizing ATP and recycling its by-product ADP as an energy source.

## Graphical Abstract

**Graphical Abstract:**
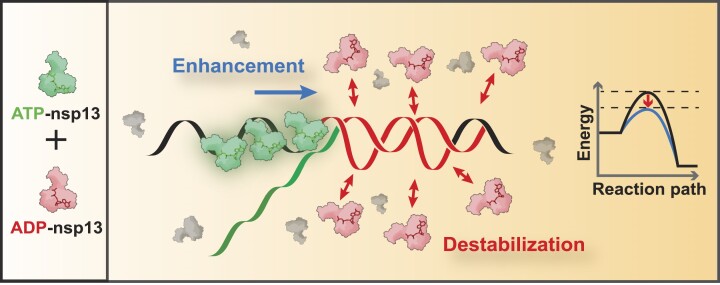

## Introduction

Helicases are nucleic acid motors that move along a single-stranded (ss) track and concomitantly separate the complementary strand from various nucleic acid duplexes during DNA replication, recombination, and repair as well as RNA transcription and degradation [1]. They achieve separation by exerting forces generated from alternating conformational stepping along their tracks, driven by repeated cycles of ATP hydrolysis [2–4]. Some helicases function as monomers but display higher unwinding activity under certain conditions in which multiple monomers are able to run together, suggesting their cooperativity with higher-order oligomeric states [5]. For example, most replicative helicases form hexameric rings to enhance processivity via topological links and to strengthen the power stroke for unwinding by firing several ATP molecules at a time [6, 7]. In this case, the hexameric structure is essential for the processive power stroke activity. Likewise, for monomeric helicases to achieve processive behaviour in several challenging conditions where a single motor is unable to provide sufficient force (e.g. secondary structures and a protein load block), multiple monomers increase the likelihood of overcoming this challenge via a vectorial physical interaction that allows a synchronized inchworm-like motion (i.e. conformational changes) coordinated by ATP hydrolysis [3].

Helicases also act as chaperones to remodel nucleic acids and nucleic acid–protein complexes by resolving secondary structures and disrupting nucleic acid–protein interactions [8]. The chaperone activities are vital for maintaining the integrity and functionality of the cellular RNA pool and the G-quadruplex (G4) of DNA and RNA. These activities ensure that nucleic acids are correctly folded and assembled into ribonucleoprotein complexes. The chaperone activity stems from the properties of helicases. For example, ATP-dependent unwinding is the primary mechanism that allows the helicase to destabilize incorrect secondary structures, enabling them refold correctly [9, 10], and free energy changes due to the binding and release of nucleic acid-interacting proteins are another source of chaperone activity that promotes correct folding [11, 12]. The cyclic binding and release facilitate the dynamic remodelling of secondary and higher-ordered structures of RNA. The distinction between helicase and chaperone activities is unclear, and how they are interconverted and linked remains to be elucidated.

The severe acute respiratory syndrome coronavirus (SARS-CoV) nsp13 protein is a 67 kDa protein and is a superfamily 1B (SF1B) helicase that unwinds double-stranded DNA (dsDNA) and dsRNA in the 5′ to 3′ direction [13–17]. The possible functional forms of nsp13 are not fully known although the SARS-CoV-2 replication–transcription complex has been reported [18–20]. The helicase essential for viral replication has been proposed to be a potential candidate for anti-SARS therapy [21]. We studied the nsp13 sequence of SARS-CoV as a model for the coordination of helicase and chaperone activities. The fundamental aspect of the two activities might be beneficial for developing antiviral drugs [22–24]. However, how ensembles of collective helicases work in concert to generate accumulative forces and catalyse laborious unwinding reactions is poorly understood. Since the rapid spread of the SARS virus can be attributed in part to the rapid rate of viral genome replication, which is a cooperative process [25], there might be unique mechanisms underlying the different structural and functional modalities involved.

We used single-molecule fluorescence resonance energy transfer (smFRET) [26] and single-molecule DNA flow-stretching (smFS) [27] assays to examine the unwinding mechanism of nsp13 on partial DNA duplexes. The assays evaluated how the concentration of nsp13 [nsp13] affects unwinding, in the presence of ATP, ADP, or both. Our kinetic analysis indicated that the unwinding rate, step size, and probability of moving forwards increase as more helicases are loaded in tandem onto and work together along the 5′ ss tail. At higher [ATP], the maximum velocity of unwinding increases in a stepwise manner by increasing the number of enzymes loaded onto a single DNA substrate as the length of the 5′ ss tail increases. This suggests that the force exerted is additive, depending on the number of motors loaded onto the substrate junctions. More importantly, we found that the novel chaperone activity of nsp13 destabilizes DNA or RNA duplexes in an ADP-dependent manner. This function was newly named ‘chaperonal *bumping* activity’, which facilitates the unwinding of duplex structures. Our findings have extended the diversity of cooperative mechanisms that account for the complexity of biological processes.

## Materials and methods

### Plasmid preparation

The pHelA12 plasmid (*nsp13 gene* containing an N-terminal 6×His tag) was a gift from Dr Kim’s laboratory [14]. For Cy3 labelling of nsp13, the (GGGGS)_3_-LPETGG sequence was inserted at the C-terminus of the *nsp13 gene* (termed *nsp13-LPETGG*) in pHelA12. The *SARS-CoV-2 nsp13 gene* from a purchased plasmid (Addgene #159614) was cloned and inserted into pET28a using BamHI (New England Biolabs) and XhoI (New England Biolabs). The *Deinococcus radiodurans RecD2 (DrRecD2)* gene in pET22b was a gift from Dr Dale Wigley’s laboratory [28]. The *Rep* gene from *Escherichia coli* was cloned and inserted into pET28a using NdeI (New England Biolabs) and XhoI.

### Protein purification and Cy3 labelling

*Nsp13* in pHelA12, *nsp13-LPETGG* in pHelA12, and *SARS-CoV-2 nsp13* in pET28a were subsequently transformed into BL21 Star (DE3) *E. coli* (Thermo Fisher Scientific), and the cells were subsequently grown in 2 l of Luria broth (LB) (Conda) supplemented with 50 μg/ml kanamycin (Sigma–Aldrich) to an OD_600_ of 0.5 at 37°C. The protein was overexpressed by induction with 0.5 mM isopropyl-β-d-1-thiogalactopyranoside (IPTG) (LPS solution) and cultivation overnight at 18°C, after which the cells were harvested by centrifugation at 5000 × *g* and resuspended in buffer A containing 25 mM Tris–HCl (pH 6.8), 500 mM NaCl, and 1 mM phenylmethane sulfonyl fluoride (Sigma–Aldrich). The cells were lysed by sonication (Sonic & Materials, flat tip) for 6 min (e.g. the pulse was repeated for 2 s on and 5 s off) and centrifuged at 30000 × *g* for 30 min. The supernatant was filtered (0.45 μm, Millex-HV) and loaded into a 5 ml HisTrap HP column (Cytiva). The fractions were eluted with gradually increasing concentrations of buffer B [25 mM Tris–HCl (pH 6.8), 500 mM NaCl, and 500 mM imidazole]. The purity of the fractions was confirmed via sodium dodecyl sulfate–polyacrylamide gel electrophoresis, after which the proteins were concentrated with a centrifugal filter (Amicon Ultra-15, 50K, Merck) and exchanged into buffer C [25 mM Tris–HCl (pH 6.8), 200 mM NaCl, and 30% glycerol]. Small volumes of purified proteins were frozen with liquid nitrogen and stored at −80°C. The concentration was measured based on the absorbance at 280 nm using a NanoDrop (Thermo Fisher Scientific).

For Cy3-nsp13, purified nsp13-LPETGG was labelled with Cy3 [29]. Briefly, a mixture containing 10 μM nsp13-LPETGG and a 25-fold molar excess of the GGK-EDA-sulfo Cy3 probe (Anygen) was reacted with 70 μM Ca^2+^-independent sortase A (Addgene #64984) in labelling buffer [50 mM Tris–HCl (pH 7.5) and 150 mM NaCl] for 1 h at room temperature. Cy3-nsp13 was further purified by using Ni-NTA agarose (QIAGEN) to remove the free Cy3 probe and sortase A. Cy3-nsp13 was eluted with buffer D [25 mM Tris–HCl (pH 6.8), 200 mM NaCl, and 500 mM imidazole], and the buffer was changed to buffer C. Small volumes of the purified Cy3-nsp13 protein were frozen in liquid nitrogen and stored at −80°C.

*DrRecD2* in pET22b was subsequently transformed into BL21 Star (DE3) *E. coli*, which was subsequently grown in 1 l of LB supplemented with 100 μg/ml ampicillin (Duchefa) to an OD_600_ of 0.4 at 37°C. The DrRecD2 protein was overexpressed by induction with 2 mM IPTG and cultivation for 3 h at 37°C. The overexpressed DrRecD2 was purified by methods similar to those described for nsp13, with slight modifications. The cells were lysed with the DrRecD2 lysis buffer [20 mM Tris–HCl (pH 7.5), 500 mM NaCl, and 10% glycerol]. The DrRecD2 proteins were eluted with DrRecD2 elution buffer [20 mM Tris–HCl (pH 7.5), 500 mM NaCl, 500 mM imidazole, and 10% glycerol]. Pure fractions were dialyzed against the DrRecD2 dialysis buffer [20 mM Tris–HCl (pH 7.5), 150 mM NaCl, and 30% glycerol] overnight at 4°C, and concentrated DrRecD2 proteins were frozen in liquid nitrogen and stored at −80°C. Rep was overexpressed and purified as previously described [30].

### smFRET set-up and unwinding assay

The smFRET measurements were performed on a custom-built prism-type total internal reflection fluorescence (TIRF) microscope (IX 71, Olympus). The fluorophore-labelled DNA substrates (e.g. *N* nt-60 bp dsDNA in Supplementary Table S1) in T50 buffer [10 mM Tris–HCl (pH 8.0) and 50 mM NaCl] were immobilized via biotin–neutravidin interactions in a chamber that was assembled with a PEGylated quartz slide and a cover slip [26]. The fluorescence emission from the fluorophores excited with a green laser (532 nm, Coherent) was measured with a UplanSApo 60× objective lens (NA = 1.20, Olympus). The fluorescence signals were separated for donors (Cy3) and acceptors (Cy5) by a dichroic mirror (660 nm cut-off) and recorded on an EMCCD camera (iXon Ultra 897, Andor) with a 0.1 s temporal resolution. The fluorescence intensities were acquired from the EMCCD camera and amplified by an EM gain. The Cy3 and Cy5 signals were selected by Gaussian fitting and signal cut-off above the average background signal. The peak positions in the Cy3 and Cy5 channels were colocalized using a mapping algorithm compiled in IDL software (ITT visual information solutions). The intensities were extracted from the peaks found on the recorded video files. The FRET efficiency (*E*_FRET_) was calculated by the equation *E*_FRET_ = (*I*_A_ − *α* × *I*_D_)/(*I*_A_+ *I*_D_), where *α* is the leakage correction factor and *I*_D_ and *I*_A_ are the intensities of the donor (Cy3) and acceptor (Cy5), respectively.

For smFRET experiments, Bio_45 nt_Cy5 and Phos_15 nt_Cy3 (Supplementary Table S2) were annealed to complementary ssDNA with tails varying in length from 0 to 90 nt and ligated by T4 DNA ligase (New England Biolabs). The DNA constructs (‘*N* nt-60 bp dsDNA’ in Supplementary Table S1) were immobilized onto the imaging chamber. The reaction was started by injection of reaction buffer containing nsp13 and ATP (or ATP and ADP) in imaging buffer containing 20 mM Tris–HCl (pH 6.8), 20 mM NaCl, 6 mM MgCl_2_, 100 μg/ml bovine serum albumin (BSA) (Thermo Fisher Scientific), 1 mg/ml 6-hydroxy-2,5,7,8-tetramethylchroman-2-carboxylic acid (Trolox) (Sigma–Aldrich), 1 mg/ml glucose oxidase (Sigma–Aldrich), 0.03 mg/ml catalase (Cayman), and 1% (v/v) dextrose (Thermo Fisher Scientific). The reaction events were recorded for 2 min to obtain real-time trajectories of enzyme dynamics, and FRET histograms were constructed to visualize the reaction state by extracting fluorescence intensities of Cy3 and Cy5. All the experiments were carried out at 37°C, and the data were analysed with MATLAB (Mathworks) and Origin (OriginLab).

### Measurements of FRET fluctuations

FRET fluctuation data were collected and quantified from the point of injection (i.e. ∼6 s from the start of recording) to the point of bleaching during the reaction. Reactions were performed with or without 150 nM nsp13 and different types of nucleotides, e.g. adenosine 5′-triphosphate (ATP) (Sigma–Aldrich), adenylyl-imidodiphosphate (AMPPNP) (Roche), or adenosine 3′,5′-diphosphate (ADP) (Sigma–Aldrich), in imaging buffer. The mean smFRET ($\bar{X}$) and standard deviation (SD) were calculated by the equation ${\mathrm{S}}{\mathrm{D}}{\mathrm{\ }} = {\mathrm{\ }}\sqrt {\frac{{{\mathrm{\Sigma }}_{i = 1}^{\mathrm{n}}{{{( {{{X}_i} - \bar{X}} )}}^2}}}{{n - 1}}}$, where *n* is the frame number.

### Binding ability assay

For the 722 bp duplex DNA substrates, 662 bp long dsDNA was amplified from the lambda DNA (New England Biolabs) by using the Bio_F_primer and the Phos_15 nt_R_primer, as shown in Supplementary Table S2. A 14 nt long 5′ overhang was created via the 3′ to 5′ exonuclease activity of T4 DNA polymerase (New England Biolabs) in the presence of deoxyadenosine triphosphate alone [31]. The product was purified by using a DNA Cleanup Kit (GeneALL). The purified DNA was annealed and ligated with Phos_linker DNA, as shown in Supplementary Table S2, to construct the first intermediate product. For the 722 bp dsDNA (Supplementary Table S1), Phos_15 nt_Alexa488 and the blunt tail DNA, as shown in Supplementary Table S2, were annealed and ligated with the first annealing and ligation intermediate products. For the binding ability assay, a 488 nm laser was used for 5 s to identify Alexa488-labelled DNA spots. To measure binding events, a reaction buffer containing Cy3-nsp13 and ATP (or ADP, or both) was added, followed by illumination with a 532 nm laser and recording for 300 s. Time trajectories of Cy3 intensity after background extraction were analysed in MATLAB and Origin.

### Duplex DNA melting temperature (*T*_m_) shift assay

First, 500 nM 15 bp dsDNA (Supplementary Table S1) was preincubated with 1× SYBR Green I (Invitrogen) in incubation buffer containing nsp13 [25 mM Tris–HCl (pH 6.8), 25 mM NaCl, 6 mM MgCl_2_, and 20 μg/ml BSA], incubation buffer containing DrRecD2 [25 mM Tris–HCl (pH 7.5), 25 mM NaCl, 6 mM MgCl_2_, and 20 μg/ml BSA], or incubation buffer containing Rep [20 mM Tris–HCl (pH 7.5), 6 mM NaCl, 1.7 mM MgCl_2_, and 20 μg/ml BSA] for 15 min at room temperature. Five hundred nanomolar protein (e.g. nsp13, DrRecD2, or Rep) was added to ADP in the corresponding incubation buffer (nsp13, DreRecD2, or Rep, respectively). The fluorescence intensity was measured by qPCR (LightCycler 480 II, Roche) with increasing temperature. The melting temperature (*T*_m_) was calculated with the equation (−Δ fluorescence/Δ temperature) (Fig. 4A).

### PAGE-based unwinding assay

Unwinding reactions were performed for 30 min at 37°C after mixing nsp13 with ATP (or ATP and ADP) in reaction buffer containing 25 mM Tris–HCl (pH 6.8), 25 mM NaCl, 6 mM MgCl_2_, 50 μg/ml BSA, 25 nM 60 nt-60 bp dsDNA (Supplementary Table S1), and 250 nM 60 nt trap DNA (Supplementary Table S2). To stop the unwinding reaction, an equal volume of quenching buffer containing 100 mM ethylenediaminetetraacetic acid (EDTA) (Bio-Rad), 20% glycerol, 0.4% SDS, and 4 U/ml proteinase K (New England Biolabs) was mixed and incubated for 10 min at 37°C. Unwinding reactions for the RNA substrate were performed in the same reaction buffer used for 60 nt-60 bp dsDNA. nsp13 was added to reaction buffer supplemented with 20 nt-20 bp dsRNA (Supplementary Table S1) and 20 nt trap RNA (Supplementary Table S2) for 10 min at 37°C, after which the unwinding reaction was stopped by adding quenching buffer. The products were separated via 12% native PAGE. The Cy5 intensity was determined by imaging with Chemi™Doc (Bio-Rad) and analysed with Image Lab (Bio-Rad).

For the DrRecD2 unwinding assay, 10 nM DrRecD2 and 1 mM ATP (or 1 mM ATP and various [ADP]) were incubated in buffer containing 25 mM Tris–HCl (pH 7.5), 25 mM NaCl, 6 mM MgCl_2_, 50 μg/ml BSA, 25 nM 10 nt-20 bp dsDNA (Supplementary Table S1), and 250 nM 20 nt trap DNA (Supplementary Table S2) for 30 min at room temperature. After the unwinding reaction was stopped by mixing the quenching buffer, unwound DNA products were resolved via 20% native PAGE. For the Rep unwinding assay, 500 nM Rep and 1 mM ATP (or 1 mM ATP and various [ADP]) were incubated in buffer containing 20 mM Tris–HCl (pH 7.5), 6 mM NaCl, 1.7 mM MgCl_2_, 50 μg/ml BSA, 25 nM 10 nt-20 bp dsDNA (3′ OH) (Supplementary Table S1), and 250 nM 30 nt trap DNA (Supplementary Table S2) for 10 min at room temperature. After the unwinding reaction was stopped by adding quenching buffer, unwound DNA products were resolved via 30% native PAGE.

### smFS assay

For the 23 kb DNA substrate, which included a 60 nt 5′ overhang (60 nt-23 kb dsDNA in Supplementary Table S1), linear lambda DNA was digested by HindIII (New England Biolabs). The 23.130 kb DNA fragments were extracted and purified using a 0.5% agarose gel and gel extraction kit (Cosmo Genetech). The purified 23.130 kb DNA fragments, Bio_60T_18 nt DNA, and Phos_linker DNA, as shown in Supplementary Table S2, were annealed and ligated in T50 buffer at a molar ratio of 1:2:2. The ligated fragments were annealed and ligated with 100-fold molar excess of Phos_7 nt_Dig, as shown in Supplementary Table S2. The purified 60 nt-23 kb dsDNA (Supplementary Table S1) was stored in small volumes at −20°C for long-term storage.

The bright field set-up was applied to the smFS assay using a UplanSApo 10× objective lens (NA = 0.40, Olympus) and sCMOS Zyla (Andor). The smFS assay was performed as described previously [32], with some modifications. The flow chamber (3.0 mm × 25.0 mm × 0.1 mm) was assembled using a functionalized cover glass with biotin-PEG and mPEG (with a mass ratio of 1:10, Laysan Bio), a Piranha cleaned quartz slide, and a double-sided adhesive sheet (Grace Bio-Labs). Both ends of the chamber were connected with polyethylene tubes (I.D 0.76 mm, O.D 1.22 mm, Becton Dickinson) to a syringe pump (Harvard apparatus) via a damper to ensure a consistent rate of buffer flow. Then, 0.2 mg/ml neutravidin in 0.5 ml of T50 buffer was added to the chamber at a rate of 0.04 ml/min, and 1 ml of the blocking buffer [20 mM Tris–HCl (pH 6.8), 50 mM NaCl, 2 mM EDTA, and 100 μg/ml BSA] was added to remove excess neutravidin (Thermo Fisher Scientific) molecules. After immobilizing the DNA substrates (∼50 pM) in a chamber with a 0.012 ml/min flow rate for 30 min, Anti-Dig (Roche)-coated Dynabeads (2.8 μm, 1 × 10^7^ beads/ml, Invitrogen) were added to the chamber for 30 min to tether the substrates to the Dig-conjugated DNA substrates. The free beads were washed with excess blocking buffer (2 ml). To protect against nonspecific interactions with the surface, magnet forces (∼1 pN) generated by a vertical rare earth magnet were calibrated via power spectrum density analysis [33]. After the chamber was equilibrated with reaction buffer [20 mM Tris–HCl (pH 6.8), 20 mM NaCl, 6 mM MgCl_2_, and 100 μg/ml BSA], the unwinding reaction was performed in reaction buffer containing nsp13 and ATP (or ATP and ADP) for 30 min at a flow rate of 0.012 ml/min. Images were acquired in 1 s by Metamorph software (Molecular Devices). The beads were precisely tracked with a DiaTrack 3.04 [34], and the data were analysed with MATLAB and Origin.

### Binding affinity assay between nsp13 and nucleotides

The binding affinity of nsp13 to mant-nucleotides (mant-ATP or mant-ADP; Jena Bioscience) was measured as previously described [35], with modifications. Briefly, nsp13 (1 μM) was incubated for 30 min with or without a five-fold molar excess of 15 bp dsDNA (Supplementary Table S1) and varying concentrations of mant-ATP or mant-ADP. The incubation was performed in a buffer containing 20 mM Tris–HCl (pH 6.8), 20 mM NaCl, 6 mM MgCl_2_, and 40 μg/ml BSA. Fluorescence measurements of mant-ATP or mant-ADP were conducted using a SPARK plate reader (Tecan) in a 96-well half-area black microplate (Greiner Bio-One, 675076). The excitation and emission wavelengths were set at 290 and 448 nm, respectively, with a gain setting of 90. Each measurement represents the average of five readings per well, derived from at least three independent experiments.

### DNA and RNA oligos

All DNA and RNA oligos were purchased from Integrated DNA Technologies or Macrogen. Amine-modified DNA or RNA strands were labelled with Alexa Fluor™ 488 NHS ester (Thermo Fisher Scientific), Cy3 NHS ester (Cytiva), or Cy5 NHS ester (Cytiva). For the assays, the corresponding ssDNA or ssRNA was annealed with complementary strands in T50 buffer by heating for 3 min at 90°C and slowly cooled to room temperature. Ligations were performed by using T4 DNA ligase when necessary. All oligo sequences and modification information are listed in Supplementary Table S2.

## Results

### smFRET assay for assessing the unwinding activity of SARS-CoV nsp13

**Figure 1.**
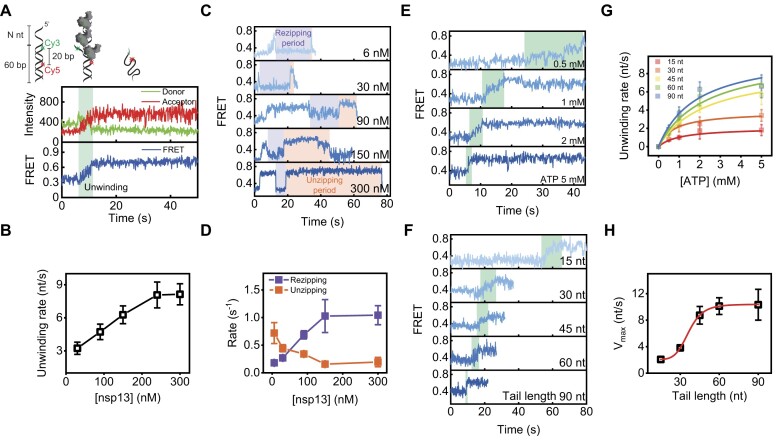
Cooperative unwinding by the ATP-driven helicase function of nsp13. (**A**) A representative FRET–time trajectory showing how the unwinding time was measured and a cartoon showing the status of DNA unwinding by nsp13 helicases. The green and red traces denote donor and acceptor intensities, respectively, and the blue trace is the FRET efficiency determined by the acceptor intensity divided by the sum of the donor and acceptor intensities. (**B**) Unwinding rate versus nsp13 concentration obtained from 90 nt-60 bp dsDNA at 2 mM ATP. (**C**) Repetitive unwinding patterns with rezipping (purple) and unzipping (orange) events at various nsp13 concentrations. (**D**) Rezipping (purple) and unzipping (orange) rates as a function of nsp13 concentration obtained from 45 nt-60 bp dsDNA at 2 mM ATP (bottom). Representative FRET–time trajectories as a function of ATP concentration (**E**) and the length of the ss-overhang tail (**F**). (**G**) Unwinding rate versus ATP concentration obtained from 60 bp dsDNA at 150 nM nsp13 with various lengths of the ss-overhang tail. Data were fitted with the Michaelis–Menten equation. Each data point represents the mean of three replicates, with each replicate representing the average of >100 molecules. The error bars denote the standard error of the mean (SEM) unless otherwise indicated. (**H**) *V*_max_ versus ss-overhang tail length, as described in panel (G). The error bars indicate the standard errors.

It has been reported that SARS-CoV nsp13 exhibits functional cooperativity, exerting unwinding activity via the synergistically interacting oligomers [14] on ss-overhangs as a function of length, similar to the effects of other helicases (NS3 and Dda) [36, 37]. Although SARS-CoV nsp13 is a retroviral helicase, it is known to be able to unwind dsRNA [38–40] and dsDNA [14, 17]. Many researchers use dsDNA substrates [3, 14] to measure nsp13 helicase activity because dsDNA is more stable and easier to handle than dsRNA, which is prone to degradation by RNases. This avoids complications from RNA breakdown and allows for a more reliable evaluation of nsp13’s unwinding capabilities. To characterize the cooperativity at the single-molecule level, we used smFRET on a partial duplex substrate containing a 5′ ssDNA tail, ranging from 15 to 90 nt, and 60 bp of DNA (Fig. 1A, top left). The FRET donor (Cy3) and acceptor (Cy5) were covalently attached onto the 60 bp duplex, Cy3 near the junction and Cy5 20 bp into the duplex, respectively. The partial duplex was immobilized on a polymer-coated surface by a biotin–neutravidin interaction, and the results were recorded by a two-colour TIRF microscope [26]. When reaction buffer containing nsp13, MgCl_2_, and ATP was added to the DNA molecules on the surface via a flow channel system [26], the 5′ tail strand was unwound, and the 3′ complementary strand with Cy3 and Cy5 coiled up (Fig. 1A, top right). This reaction separated the dsDNA into two ssDNAs, resulting in a FRET increase due to a decrease in the time-averaged distance between the two fluorescent dyes. A representative FRET–time trajectory (Fig. 1A, top) displays an initial FRET value at *E*∼ 0.3 before the reaction and a final FRET value at *E*∼ 0.6 after the reaction.

### Functional cooperativity of the ATP-driven nsp13 helicase characterized by smFRET

We examined the dependence of the nsp13 concentration [nsp13] and found that the unwinding rate increased as a function of [nsp13] (Fig. 1B). FRET–time trajectories (Fig. 1C) revealed repetitive unwinding activity at low [nsp13] but processive unwinding activity at high [nsp13]. To characterize the [nsp13]-dependent repetitive behaviour, we analysed two periods: (i) the rezipping period (Fig. 1C, purple), during which FRET stayed at low FRET states due to the high rezipping tendency of DNA (i.e. weak nsp13 unwinding activity and intrinsic annealing into the duplex); (ii) and the unzipping (i.e. unwinding) period (Fig. 1C, orange), during which FRET stayed at high FRET states due to the high unwinding tendency of nsp13 (i.e. strong nsp13 unwinding activity). Quantification (Fig. 1D) revealed that the repetitive behaviour of nsp13 decreased but that its processing behaviour increased as a function of [nsp13], similar to what has been observed for other nonhexameric helicases [3, 41, 42]. When the ATP concentration [ATP] and 5′ ss-loading tail length of the DNA substrate increased, the population of unwound DNA increased, as shown by the individual trajectories (Fig. 1E and F) and ensemble distributions (Supplementary Fig. S1). The maximum unwinding rate (*V*_max_) increased with increasing tail length (Fig. 1G and H), indicating multimeric cooperativity. A plot of the *V*_max_ versus tail length showed a clear sigmodal curve, suggesting cooperativity, presumably according to the training model reported for other helicases [36, 37, 43]. Briefly, when one helicase unwinds a double-stranded (ds) nucleic acid, the growing portion of the unwound strand recruits more helicases. These multiple helicases work in a coordinated fashion, with each helicase functioning as a separate entity but interconnected with the others to efficiently unwind the ds nucleic acid.

The unwinding rate of nsp13, measured by gel-based assays, has been reported in a wide range from ∼1 to ∼50 bp/s [15, 16, 39, 44, 45], depending on the type of substrates. The rate of nsp13 obtained by smFRET on the 60 bp DNA substrate was ∼8 bp/s (Fig. 1B and G), and the value was somewhat on the slow side of that range. To confirm this again, we performed gel-based assays on a 20 nt-20 bp dsDNA and a 60 nt-60 bp dsDNA (Supplementary Fig. S2). The unwinding activity of nsp13 on the 20 nt-20 bp DNA (53.42 ± 5.14 bp/s) was ∼5.5-fold faster than that on the 60 nt-60 bp DNA (9.78 ± 1.92 bp/s), suggesting a duplex length dependence of the unwinding activity. This range of nsp13 unwinding rates obtained by the gel-based assays is comparable to the range reported for other SF1 helicases [46] and is consistent with previously characterized unwinding kinetics of nsp13 [16, 39].

### ADP-directed chaperone activity of nsp13 with unprecedented melting ability

**Figure 2.**
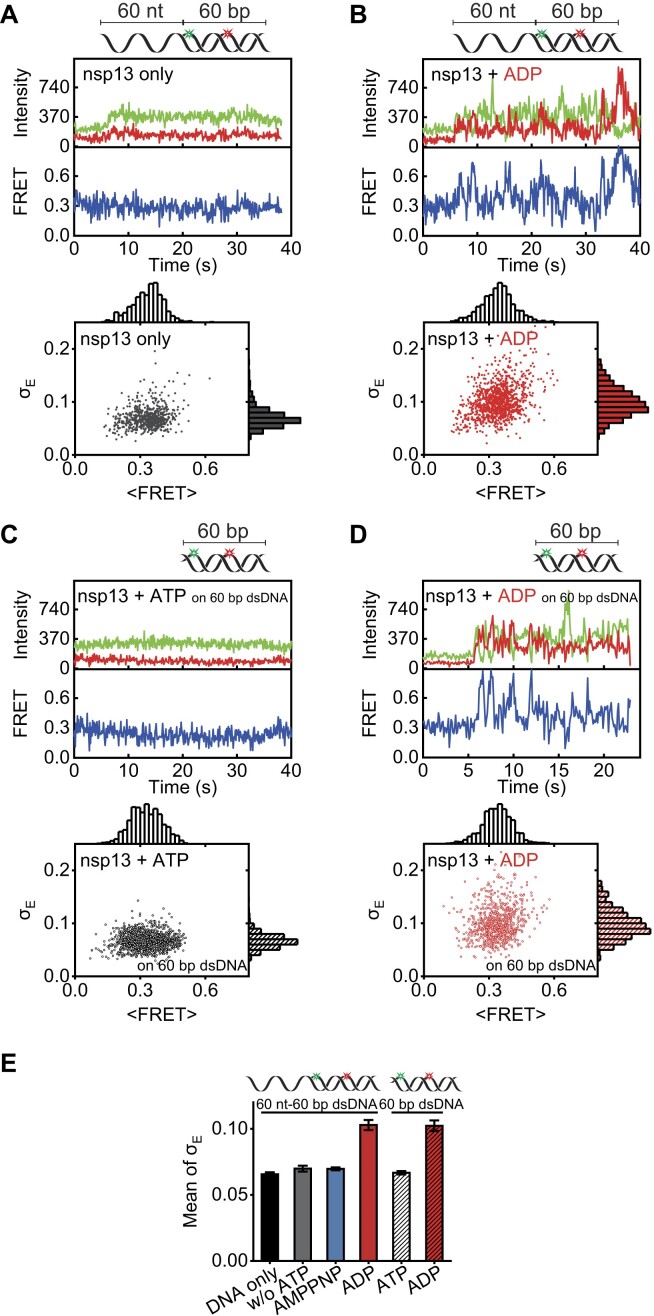
Chaperone activity of nsp13 with ADP-dependent melting capability. (**A**) Little FRET fluctuation (top) and σ_E_ versus <FRET> (bottom) were observed in the presence of nsp13 only. Here, σ_E_ is the SD and <FRET> is the mean FRET. (**B**) Large FRET fluctuation (top) and σ_E_ versus <FRET> (bottom) were observed in the presence of nsp13 and ADP. Same as in panels (A) and (B) but for 60 bp dsDNA (Supplementary Table S1) under the conditions of nsp13 and ATP (**C**) and nsp13 and ADP (**D**). (**E**) Mean σ_E_ for FRET fluctuations at 150 nM nsp13 obtained with different nucleotide variants at 2 mM. Each data point represents the mean of three replicates, with each replicate representing the average of >100 molecules. The error bars denote the SEMs unless otherwise indicated.

Recent studies have reported that the binding of ADP to nsp13 results in a large conformational change and increased surface area in the ssRNA binding region (residues 512–542), facilitating the binding of ssRNA [47, 48]. We tested how nsp13 interacts with a 60 nt ss-overhanging 60 bp dsDNA (termed 60 nt-60 bp dsDNA) in the presence of ADP, with Cy3 and Cy5 labelling sites near the ss–ds junction and 20 nt inside the duplex, respectively (Fig. 2A and B). Surprisingly, compared with those of nsp13 alone without ATP or ADP (Fig. 2A), representative FRET–time trajectories in the presence of ADP exhibited significant FRET fluctuations (Fig. 2B, top) with greater SDs (σ_E_, bottom). The higher FRET fluctuations σ_E_ were attributed to transient and partial melting of the duplex in the FRET region between the two fluorophores. A comparison of the results obtained with other nucleotide analogues suggested that the melting ability of the nsp13 was highly ADP specific (Fig. 2 and Supplementary Fig. S3), regardless of the presence of an ss-overhang in the dsDNA (Fig. 2). It is known that blunt-ended dsDNA (termed 60 bp dsDNA) does not undergo unwinding even in the presence of ATP unless a 5′ overhang is attached (Fig. 2C). Significant FRET fluctuations, however, were observed in the presence of ADP (Fig. 2D and Supplementary Fig. S4), while no such fluctuations occurred in the presence of ATP (Fig. 2A). Notably, these fluctuations were strongly dependent on [ADP] (Fig. 2E and Supplementary Fig. S5). These data suggested that the nsp13 helicase possesses an ADP-specific chaperone function that alters the stability of nucleic acid substrates, similar to the ATP-dependent chaperone function reported for other helicases [1, 9, 10].

**Figure 3.**
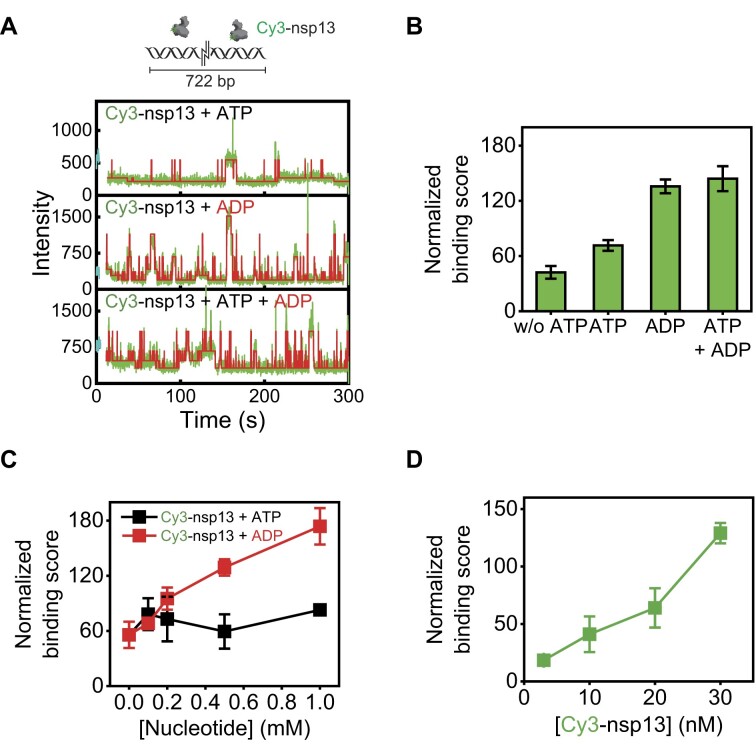
ADP empowers the binding ability of nsp13 to duplex regions of nucleic acid. (**A**) Binding of Cy3-labelled nsp13 to long DNA substrates in the presence of various nucleotide analogues. Representative intensity-time trajectory showing the binding propensity of Cy3-labelled nsp13 to 722 bp dsDNA. (**B**) The binding score was calculated by integrating the fluorescence intensity over time. (**C**) Normalized binding score versus concentration of nucleotides, calculated at 30 nM Cy3-nsp13. (**D**) The binding score was normalized to the concentration of Cy3-nsp13 in the presence of 0.5 mM ADP. Each data point represents the mean of three replicates, with each replicate representing the average of >100 molecules. The error bars denote the SEMs unless otherwise indicated.

Next, we used fluorescently labelled nsp13 to directly examine its ability to bind to a longer substrate (Fig. 3A, top) with 722 bp dsDNA (termed 722 bp dsDNA). The fluorescent nsp13 was site-specifically labelled at the C-terminus with Cy3 (termed Cy3-nsp13) by introducing a sortase recognition site (LPETGG) [29]. As a result, the activity of Cy3-nsp13 was comparable to that of wild-type nsp13 (Supplementary Fig. S6). The binding of Cy3-nsp13 to the 722 bp dsDNA was determined from fluorescence intensity time trajectories (Fig. 3A). Binding scores (i.e. propensity) were evaluated by integrating the fluorescence intensity over time and were notably greater in the presence of ADP alone or in combination with both ATP and ADP than in the presence of ATP alone (Fig. 3B). We further examined the binding score as a function of [ADP] (red in Fig. 3C), [ATP] (black in Fig. 3C) or [nsp13] (Fig. 3D). This further examination exhibited an [ADP]- and [nsp13]-dependent interaction to the dsDNA that facilitated the duplex melting.

**Figure 4.**
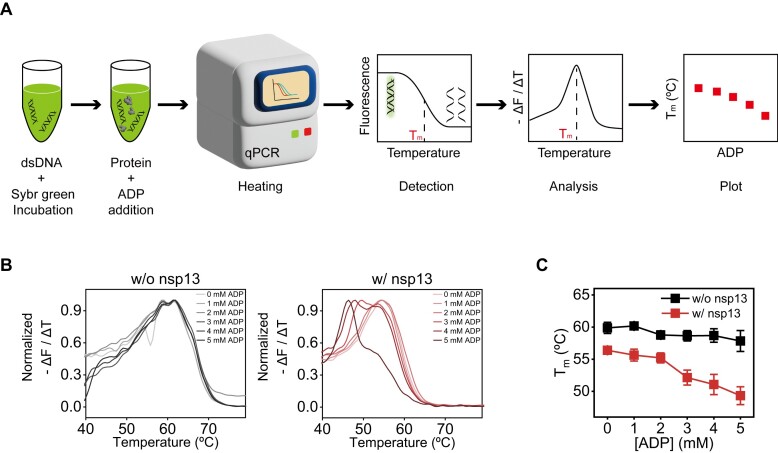
ADP-dependent destabilization of dsDNA by nsp13 lowers the melting temperature (*T*_m_) of dsDNA. (**A**) Schematics of the melting temperature (*T*_m_) shift assay. After the mixture was incubated with a mixture of duplex DNA and DNA-intercalating SYBR Green I, the proteins (nsp13, DrRecD2, or Rep) and ADP were added. Then, using qPCR, the melting temperature (*T*_m_) was measured by heating the mixture and measuring the change in fluorescence with increasing temperature. A plot of the negative derivative of fluorescence versus temperature (– Δ*F*/Δ*T*) versus temperature shows the melting temperature (*T*_m_) at the peak corresponding to the transition from dsDNA to ssDNA. Finally, the melting temperatures (*T*_m_) determined from the peaks were plotted as a function of ADP concentration. The experiments utilized 15 bp dsDNA (Supplementary Table S1), and the detailed methods are provided in the ‘Materials and methods’ section. (**B**) Representative normalized plots of Δ*F*/Δ*T* as a function of ADP concentration from 0 to 5 mM, with 500 nM nsp13 (right) and without nsp13 (left). (**C**) Melting temperature (*T*_m_) shift assay as a function of ADP concentration for 15 bp dsDNA. *T*_m_ decreases as a function of ADP concentration in the absence (black) or presence (red) of 500 nM nsp13. Each data point represents the mean of three replicates. The error bars denote the SEMs unless otherwise indicated.

To ensure duplex destabilization by ADP-loaded nsp13 (termed ADP–nsp13), we directly measured the melting temperature (*T*_m_) by a *T*_m_ shift assay [49] using 15 bp dsDNA as a function of [ADP] (Fig. 4A). In the absence of nsp13, the shift in *T*_m_ did not occur with increasing [ADP] (Fig. 4B, left), whereas in the presence of nsp13, the shift in *T*_m_ markedly decreased with increasing [ADP] (Fig. 4B, right). Overall, the data suggested that the ADP–nsp13 helicase complex transiently associate and dissociate from the ds region of blunt-ended dsDNA and induce partial DNA melting (Fig. 2B and D) through intermittent short protein−DNA interactions (Fig. 3A and B). Here, we newly named this novel behaviour ‘*chaperone-bumping* activity’, which reorganizes the thermodynamic stability of nucleic acid substrates by altering their temporal conformations for ADP-specific partial melting (Fig. 2B and D).

### Cooperative unwinding by ATP-driven helicase and ADP-directed chaperone activities

**Figure 5.**
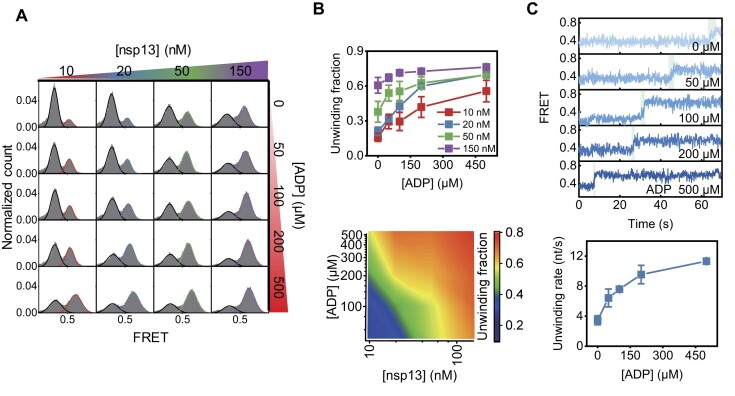
The unwinding activity of nsp13 is enhanced in the presence of ATP and ADP. (**A**) smFRET histograms 2 min after the unwinding reaction obtained from 60 nt-60 bp dsDNA at 1 mM ATP as a function of the nsp13 and ADP concentrations. Before the reaction, the histogram peaked at ∼0.3 FRET, but after the reaction, it peaked at ∼0.6 FRET, corresponding to the unwound product. The peaks of the histograms are fitted with a Gaussian distribution. (**B**) Unwinding fraction versus ADP concentration at various nsp13 concentrations (top). 2-Dimensional contour plot as in panel (A) (bottom). (**C**) Representative FRET–time trajectories as a function of ADP concentration at 20 nM nsp13 and 1 mM ATP (top). Unwinding rate versus ADP concentration (bottom). Each data point represents the mean of three replicates, with each replicate representing the average of >100 molecules. The error bars denote the SEMs unless otherwise indicated.

Based on these results (Figs 1–4), we hypothesized that ADP-dependent chaperone activity may play an important role in the unwinding activity of nsp13. We therefore tested whether the unwinding activity changes as a function of [ADP]. Above ∼2 mM ATP, the unwinding rate is saturated and very fast (Fig. 1G). We performed additional tests and found that the enzyme activity is the most sensitive and optimal for observation at ∼1 mM ATP near the *K*_m_. Quantitative measurements by smFRET showed that the presence of ADP at concentrations up to 500 μM increased the unwinding activity over a wide range of [nsp13] concentrations, from 10 to 150 nM (Fig. 5A and B). These data suggested that the unwinding activity of the helicase, which is enhanced by chaperone function, depends not only on [ADP] but also on [nsp13], presumably due to the complex formation of ADP–nsp13, also consistent with Fig. 3C and D. The analysis of individual trajectories further elucidated enhanced unwinding rates with increasing [ADP] (Fig. 5C).

**Figure 6.**
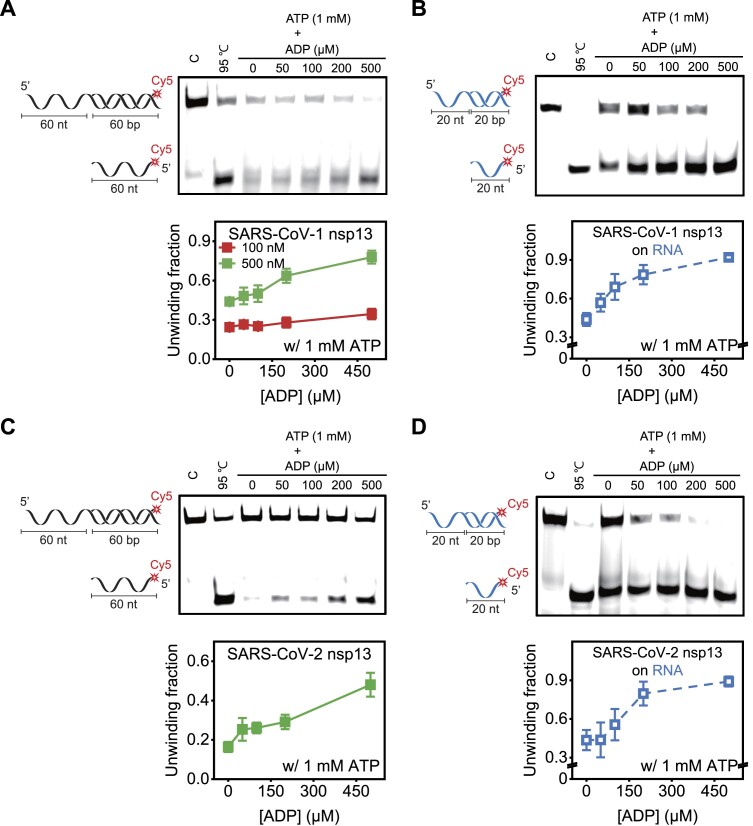
The addition of ADP enhances the helicase activity of SARS-CoV-1 and SARS-CoV-2 nsp13 on both DNA and RNA substrates in an [ADP]-dependent manner. (**A**) A gel-based unwinding assay for 60 nt-60 bp dsDNA (left cartoon and Supplementary Table S1) by 500 nM nsp13 at 1 mM ATP and increasing concentrations of ADP. Lane 1 represents the free substrate. Lane 2 shows the product heated at 95°C for 10 min. Lanes 3–7 show the unwinding reaction at a given ADP concentration (top). Quantification of the unwinding fraction at nsp13 concentrations of 100 nM (left in Supplementary Fig. S7) and 500 nM (top and right in Supplementary Fig. S7) (bottom). The ATP effect was more pronounced at higher concentrations of nsp13. (**B**) A gel-based unwinding assay for 20 nt-20 bp dsRNA (left cartoon and Supplementary Table S1) at 10 nM nsp13 and 1 mM ATP with increasing concentrations of ADP (top). Quantitative analysis of unwinding fraction as function of ADP concentration (bottom). (**C**) Same as in panel (A) but with 500 nM SARS-CoV-2 nsp13 instead of 500 nM SARS-CoV-1 nsp13. (**D**) Same as in panel (B) but with SARS-CoV-2 nsp13 instead of SARS-CoV-1 nsp13. Each data point represents the mean of three replicates. The error bars denote the SEMs unless otherwise indicated.

To validate the [ADP]-dependent chaperone activity of nsp13 on both DNA and RNA substrates, gel-based unwinding assays were employed. A notable increase in the unwinding activity was observed in the presence of 0–500 μM ADP (Fig. 6A). A comparison of the data showed that the effect of ADP was much more noticeable at higher concentrations of nsp13 (500 nM) than at 100 nM (Fig. 6A and Supplementary Fig. S7). Control experiments showed that ATP hydrolysis is essential for the unwinding activity of nsp13 (Supplementary Fig. S8). An ADP-dependent boosting effect on nsp13 was also common for dsRNA (Fig. 6B).

The data thus far are representative of SARS-CoV-1 nsp13 but not of SARS-CoV-2 nsp13. The sequences differed by only one amino acid, I570V, which is not a conserved residue. Nevertheless, the unwinding cooperativity of SARS-CoV-2 nsp13 was tested as a function of [ADP]. Consistent with this, SARS-CoV-2 nsp13 exhibited a similar increase in unwinding activity in an ADP-dependent manner for both DNA (Fig. 6C) and RNA substrates (Fig. 6D), suggesting that the two proteins are functionally almost identical.

**Figure 7.**
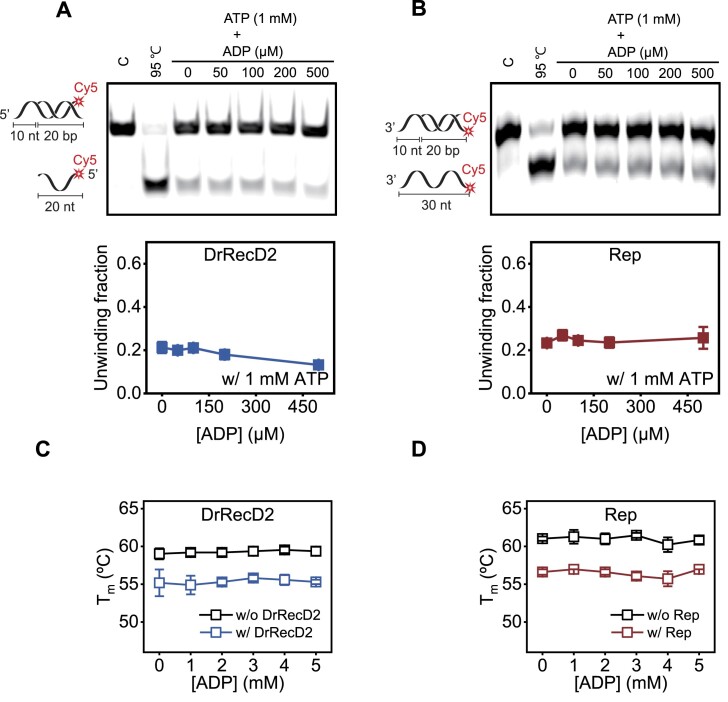
The enhancement of nsp13 unwinding activity by ADP addition is unique to SARS-CoV nsp13. Representative native PAGE images of the unwinding reaction for 10 nM DrRecD2 (**A**) and 500 nM Rep (**B**) helicase (top). The 10 nt-20 bp dsDNA sequence (Supplementary Table S1) was used to compare the activities of various helicases. The lower bands correspond to the unwound products of 20 nt ssDNA (i.e. complementary strand, A) or 30 nt ssDNA (i.e. overhang strand, B). Unwinding fraction versus [ADP] for DrRecD2 (A) and Rep (B) helicase (bottom). DNA *T*_m_ shift assays as a function of ADP concentration for 500 nM DrRecD2 (**C**) and 500 nM Rep (**D**). Each data point represents the mean of three replicates. Each data point represents the mean of three replicates. The error bars denote the SEMs unless otherwise indicated.

Next, we also examined whether the chaperone ADP–nsp13 is common for other SF1A and SF1B helicases. We selected the 3′–5′ Rep from *E. coli*and 5′–3′ RecD2 from *Deinococcus radiodurans* (DrRecD2) as representative SF1A and SF1B helicases, respectively. The gel-based and *T*_m_ shift assays showed no ADP-dependent enhanced unwinding (Fig. 7A and B) and no changes in *T*_m_ (Fig. 7C and D), indicating that the ADP-chaperone activity is unique to SARS-CoV.

### The smFS assay reveals enhanced unwinding via the cooperation of an ATP-driven helicase and an ADP-dependent chaperone

To monitor the cooperativity of the unwinding activity spatiotemporally over a large region along dsDNA, smFS was performed under a laminar flow drag force applied to a magnetic bead [27]. For smFS, one side of the 23 kb paired DNA sequence was immobilized on the surface via biotin–neutravidin interactions, and the other side was coated with digoxigenin (Dig) and connected to an anti-Dig magnetic bead (Supplementary Fig. S9). The number of nucleotides unwound by the nsp13 helicase could be determined by a change in bead position resulting from enzymatic conversion of dsDNA to ssDNA [50]. First, the unwinding activity was examined as a function of [nsp13]. Representative time trajectories and full data analysis showed that [nsp13]-dependent unwinding occurred (Supplementary Fig. S9B and C), consistent with the results of smFRET (Fig. 1B). We attribute the dependence on [nsp13] to the fact that multiple helicases can separate dsDNA more quickly and efficiently than single helicases can. This is because over time, more helicases bind to the unwound strand, functioning as a serially linked group and maximizing unwinding efficiency.

To scrutinize via smFS the enhancing effect of ADP on the slow unwinding activity in the presence of 50 nM nsp13, 1 mM ATP, and 0.5 mM ADP, the unwinding activity was observed over a wide region at the 23 kb level in DNA. The representative time trajectories of ATP alone (grey) and both ATP and ADP (red in Supplementary Fig. S9D) exhibited significant differences in the number of nucleotides unwound over time. For analysis, the average unwinding rate was determined as the number of nucleotides unwound divided by the time taken (i.e. the slope as in Supplementary Fig. S9D). Histograms (Supplementary Fig. S9E) and mean values of average unwinding rates (Supplementary Fig. S9F) revealed prominent differences in the presence of the same ATP concentration, with and without ADP (*n* = 411 and 242, respectively). In addition, the histogram and mean values for 1 mM ATP and 0.5 mM ADP were very similar for 2 mM ATP (*n* = 411 and 407, respectively).

### Model of cooperative unwinding by the dual mode of action of nsp13

**Figure 8.**
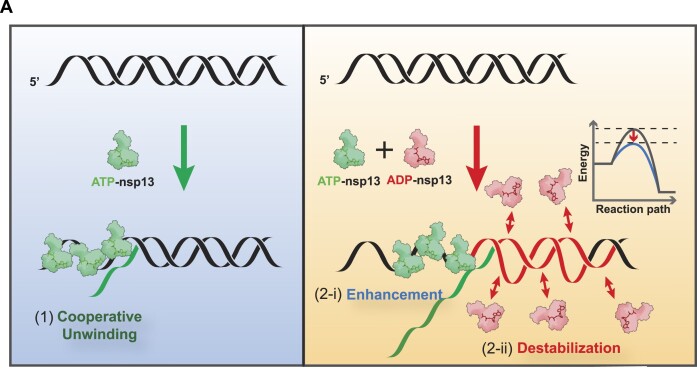
Cooperative unwinding model of nsp13 with dual modes of action. (**A**) Functional cooperativity of collective helicases, similar to the train-like model (first mode). Multiple helicases form a train-like chain during the unwinding reaction, with an increasing number of helicases landing on the unwinding strand and working cooperatively (left). nsp13 can form ADP–nsp13 complexes in solution; these complexes utilize the chemical byproduct of ATP (i.e. ADP) to perform transient but rapid association and dissociation and function to melt portions of dsDNA (second mode). Overall, nsp13 utilizes dual modes of action to expedite synergistic unwinding reactions: The first mode (2-i, right) augments the physical force to unwind its substrates through the cooperation of multiple motor proteins (i.e. ATP-driven helicase activity), whereas the second mode (2-ii, right) diminishes the energy barrier of the reaction by destabilizing dsDNA (i.e. ADP-directed chaperone activity).

Previous studies have demonstrated that nsp13 exhibits functional cooperativity, wherein multiple helicases concurrently act on the gradually unwinding strand during the unwinding reaction (Fig. 8, left). This mode of functional cooperativity was formerly termed the ‘train-like’ action [14, 37, 43] due to its resemblance to train behaviour (designated the first mode). However, our investigation revealed an exceptionally unique melting capability of SARS-CoV nsp13, which utilizes the chemical by-product of ATP (i.e. ADP). In conjunction with the train-like mode, nsp13 can form ADP–nsp13 complexes in solution, which undergo transient but rapid association and dissociation kinetics that interact with the downstream portion of dsDNA in an [ADP–nsp13]-dependent manner (designated the second mode). This second mode interaction between the ADP–nsp13 complex and dsDNA serves to lower the energy barrier of dsDNA melting (Fig. 8, right), similar to chaperone activity. Overall, nsp13 utilizes a dual mode of action to expedite the synergistic unwinding reaction. First, the physical force required to unwind substrates is augmented by the cooperation of multiple motor proteins (i.e. ATP-driven helicase activity). Second, the energy barrier of the reaction is diminished by destabilizing dsDNA (i.e. ADP-directed chaperone activity).

## Discussion

There are no reports showing that chaperone function is activated when ADP nucleotides are loaded onto SARS-CoV nsp13, but nsp13 can acquire chaperone function by forming an ADP–nsp13 complex even at ∼0.5 mM ADP, which is a physiologically relevant concentration. In fact, chaperone activity alone cannot unwind dsDNA (Supplementary Fig. S8), but ADP–nsp13 in solution performs transient binding and unbinding on the substrate with unprecedented melting ability (Fig. 2B and D), helping the primary unwinding reaction of the ATP hydrolysis-driven helicase. While this chaperone function is less critical at high ATP concentrations, its activation can enhance the rate to near-maximum levels (Supplementary Fig. S10), when ATP concentrations drop below the *K*_m_ value (e.g. 1 mM). Notably, the chaperone activity was dependent on [ADP] even under stringent conditions (i.e. high ionic strength at 100 mM NaCl), ruling out the possibility of nonspecific binding of nsp13 (Supplementary Fig. S11). Taken together, these findings indicate that the nsp13 helicase performs spatiotemporal coordination between ATP-driven motor and ADP-loaded chaperone functions. In space, the nsp13 helicases bind in series along the ss-loading tail to unwind the ss–ds fork junction, while the ADP–nsp13 complex in solution destabilizes the substrate. In contrast, the same nsp13 helicase has two different modes, and these can work together to synchronously unwind substrates during vectorial force integration over time by utilizing ATP and ADP in different nucleotide states.

We measured the binding affinity of mant-nucleotides (mant-ATP or mant-ADP) to nsp13 [35]. The binding affinity of ATP to nsp13 was ∼2.5 times higher than that of ADP, which is consistent with the ATP hydrolysis process of nsp13 [47]. However, in the presence of excess dsDNA (five-fold relative to the concentration of nsp13), the affinity of nsp13 for ADP increased significantly, while no substantial change was observed in its affinity for ATP. These findings suggest that the interaction among ADP, nsp13, and dsDNA induces a notable alteration in nsp13’s binding affinity for ADP, as depicted in our proposed model (Supplementary Fig. S12). From a biological perspective, during rapid viral infection, ATP depletion likely leads to the accumulation of ADP, facilitating ADP binding to nsp13, and enabling it to perform its function. This effect is particularly pronounced in the presence of dsDNA. Currently, it is unclear whether ADP binds the same site as ATP within the active site of nsp13. Addressing it will require detailed mutagenesis experiments and further testing. Future studies in this area could provide critical insights into the unwinding mechanism of nsp13.

This cooperativity due to the bimodal nature may be important for the rapid spread of the SARS virus. For example, after viral infection, viral replication is very active, and ADP is rapidly generated as a byproduct of ATP consumption. As a result, the viral helicase forms an ADP–nsp13 complex in solution and activates its function as a new chaperone, destabilizing dsDNA in an [ADP]-dependent manner (Figs 3C and 4C). In other words, even if [ATP] is decreased due to local ATP depletion, the replication delay of the viral genome can be prevented by chaperone activity. This may be an important property that may aid the physiology of acute coronaviruses, e.g. MERS-CoV and SARS-CoV. Although the nsp13 forms a complex with the RNA-dependent RNA polymerase (subunits nsp7/nsp8_2_/nsp12) [18, 20], it is believed to play important roles in many aspects of the viral life cycle, including mRNA capping [51, 52], viral RNA stability [16], translation [53], and replication [39]. Since free nsp13 helicases can interact with overall virus genomic and subgenomic RNA, the destabilization of nucleic-acid double helices by ADP–nsp13 may accelerate viral metabolic reactions and thus be conducive to rapid virus replication and release kinetics.

Our report is a novel example of the ADP-dependent chaperone activity of the helicase superfamily, which expands the functional divergence of helicases through sequence evolution to achieve enhanced cellular activity. The continued emergence of current coronavirus variants and the resulting ineffectiveness of vaccines are important tasks facing humanity, and we hope that our discovery of the unique property of SARS-CoV will contribute to the development of effective treatments.

## Supplementary Material

gkaf034_Supplemental_File

## Data Availability

The data underlying this article will be shared on reasonable request to the corresponding author.
