# Supplementary material for: A novel ADP-directed chaperone function facilitates the ATP-driven motor activity of SARS-CoV helicase
Source: Nucleic Acids Res. 2025 Jan 29;53(3):gkaf034. doi: 10.1093/nar/gkaf034 (PMC11775617; doi:10.1093/nar/gkaf034)
Supplement: gkaf034_Supplemental_File [file gkaf034_supplemental_file.pdf]

SUPPLEMENTARY DATA

A novel ADP-directed chaperone activity facilitates the ATP-driven motor activity of SARS-CoV helicase

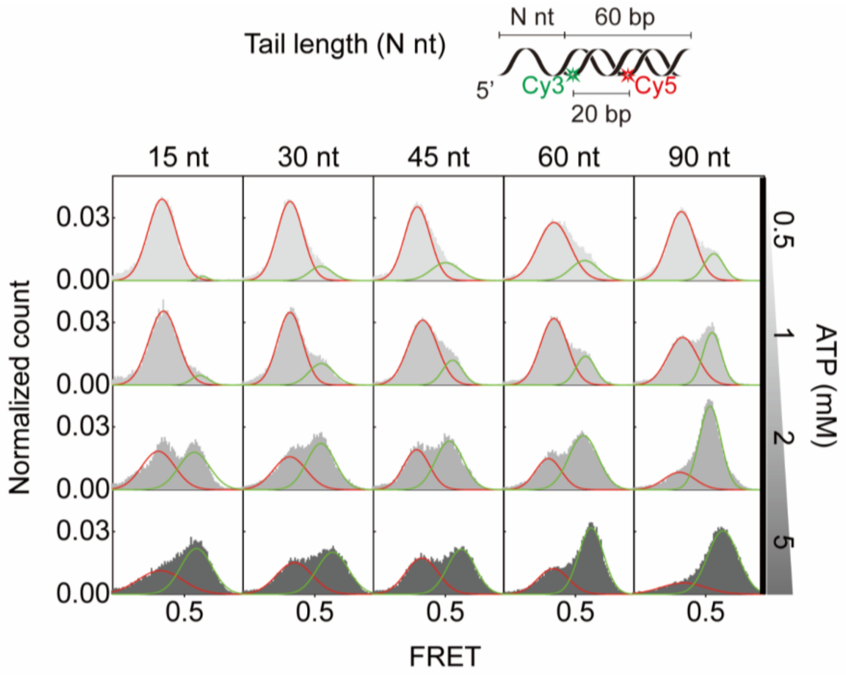

Supplementary Figure S1. Unwinding activity of nsp13 as a function of the 5′ tail length of the DNA substrate and ATP concentration.

smFRET histograms 2 min after the unwinding reaction as a function of tail length (15, 30, 45, 60, and 90 nt from left to right) of DNA substrate and ATP concentration (0.5, 1, 2, and 5 mM from top to bottom) at 150 nM nsp13. The top-right illustration represents the DNA substrate employed in the experiment (N nt-60 bp dsDNA in Supplementary Table S1). Histograms were generated from multiple data points at different positions after 2 min of the unwinding reaction. The population of unreacted DNA substrates peaked at low FRET (E_{FRET} = ~0.3), while the population of unwound DNA substrates peaked at high FRET (E_{FRET} = ~0.6). Histograms were fitted with a Gaussian distribution (red and green lines, respectively).

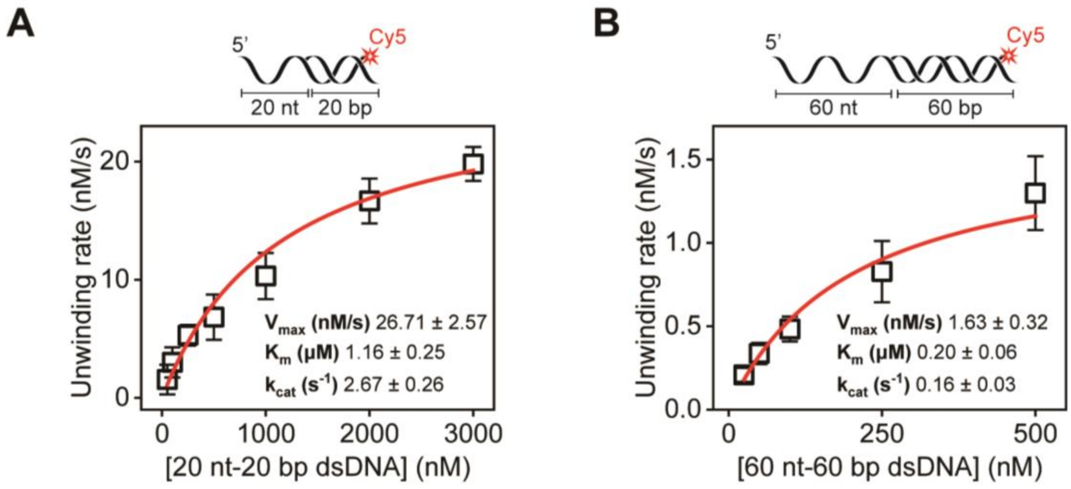

Supplementary Figure S2. Dependence of the unwinding rate of nsp13 on substrate length.

(**A, B**) Initial substrate unwinding rates (nM/s) of nsp13 depending on the concentration of DNA substrates, 20 nt-20 bp dsDNA (A) and 60 nt-60 bp dsDNA (B). Gel-based unwinding reactions were performed under the condition of 10 nM nsp13 and 5 mM ATP. Each data point represents the mean of more than three replicates. The error bars denote the standard error of the means (SEMs). The values of V_{max}, K_m and k_{cat} obtained from the Michaelis-Menten model fitting (red line) were inserted into each plot. The unwinding rates of nsp13, determined from k_{cat} values, were 53.42 ± 5.14 bp/s for 20 nt-20 bp dsDNA and 9.78 ± 1.92 bp/s for 60 nt-60 bp dsDNA.

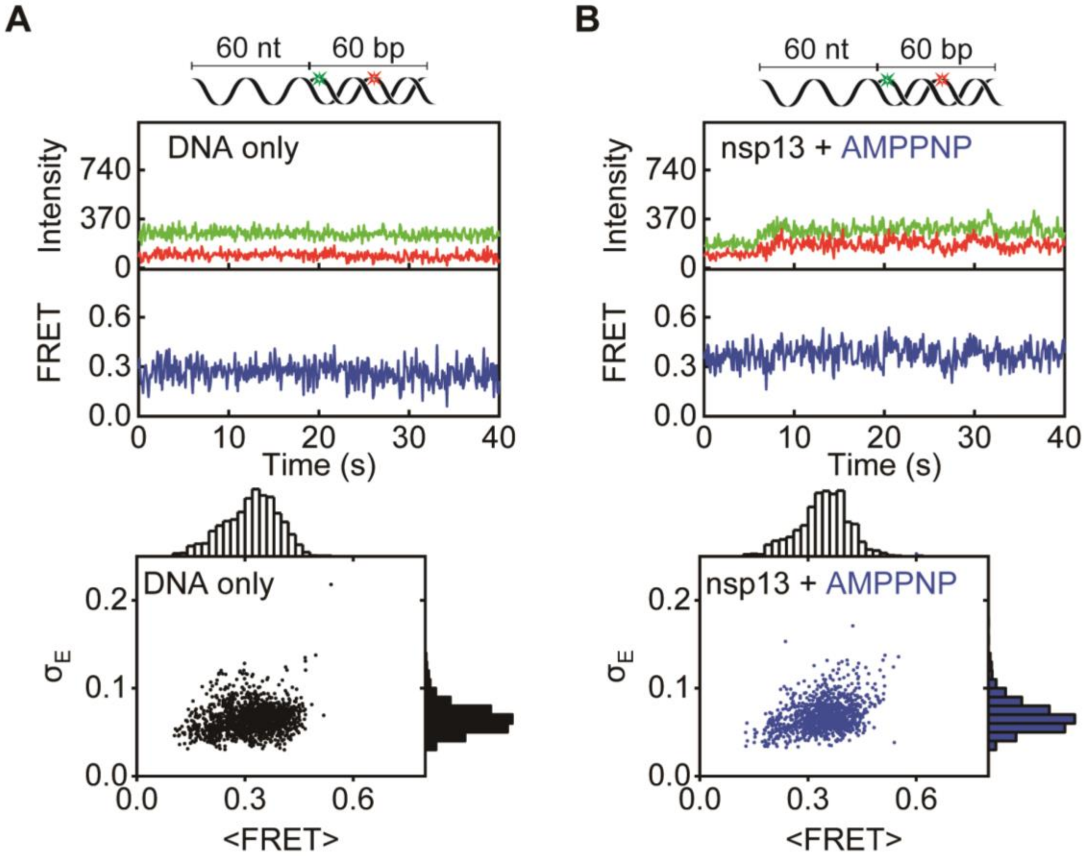

Supplementary Figure S3. FRET fluctuation assay of DNA alone, with nsp13 and AMPPNP as control experiments.

(**A, B**) Representative Cy3 (green) and Cy5 (red) intensity profiles and FRET (blue) trajectories obtained from 60 nt-60 bp dsDNA (Supplementary Table S1) with DNA alone (A) or in the presence of 150 nM nsp13 and 2 mM AMPPNP (B) (top). A scatter plot of σ_E (S.D.) versus < FRET > (mean of FRET) obtained from the equation (see Material and Methods) for individual FRET-time trajectories (top panels) (bottom). σ_E reflects the amplitude of the fluctuations.

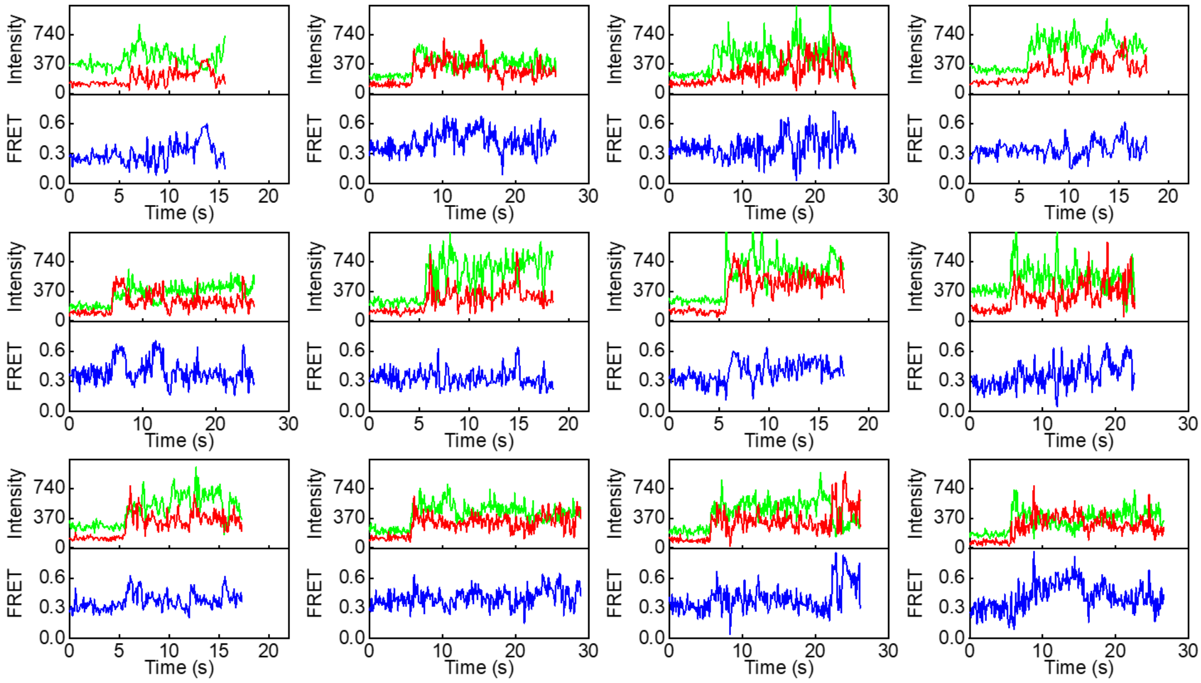

Supplementary Figure S4. Representative FRET-time traces in the presence of nsp13, ADP, and Mg^{2+}.

Most FRET-time traces in the presence of nsp13, ADP, and Mg^{2+} displayed ADP-specific fluctuations, as illustrated by the red scatter in the lower panel of Fig. 2D

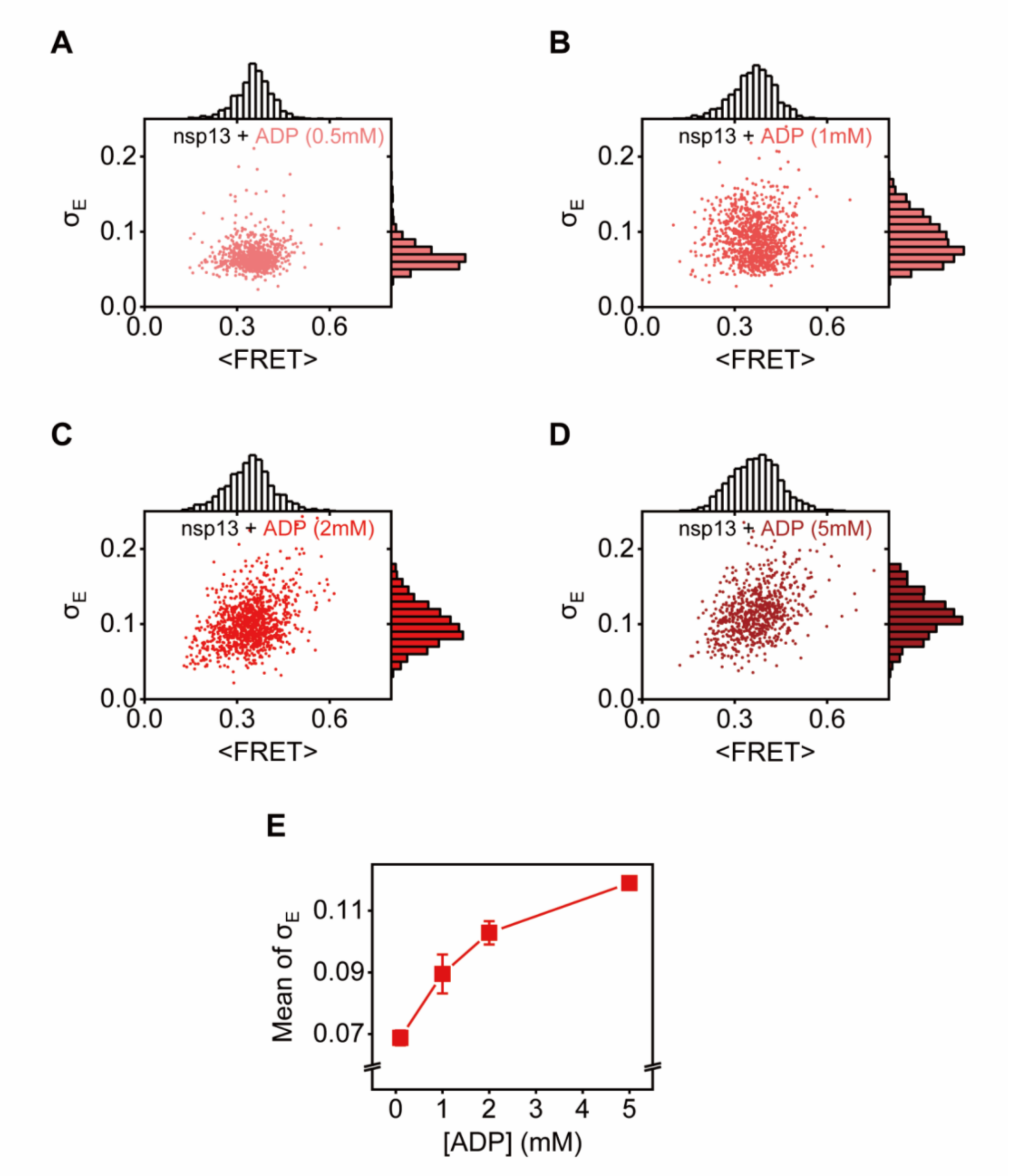

Supplementary Figure S5. FRET fluctuation assay with nsp13 as a function of ADP concentration.

(**A-D**) A scatter plot of σ_E versus < FRET > with 150 nM nsp13 and various [ADP] values at 0.5 mM (A), 1 mM (B), 2 mM (C), and 5 mM (D). (**E**) A plot of σ_E versus [ADP]. Each data point represents the mean of more than three replicates. The error bars denote the SEMs.

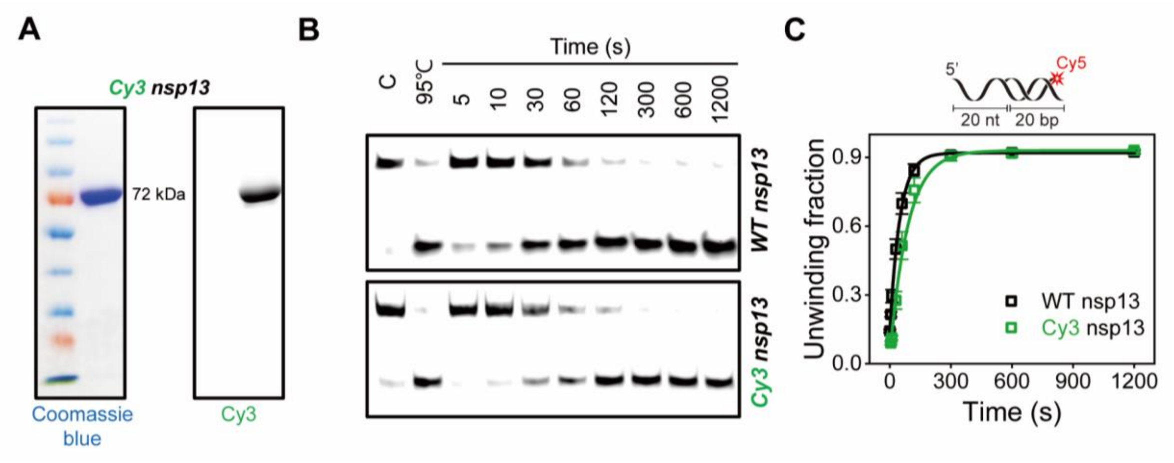

Supplementary Figure S6. The unwinding activity of Cy3-labelled nsp13 is comparable to that of native nsp13.

(**A**) SDS PAGE gel images for Cy3-labelled nsp13 with Coomassie blue staining (left) and Cy3 fluorescence intensity (right). The labelling efficiency of protein is approximately 45%, as determined by the ratio of the Cy3 concentration to the protein concentration using a Nanodrop. (**B**) Representative native PAGE images of the unwinding assay for 20 nt-20 bp dsDNA (Supplementary Table S1) with nsp13 (top) and Cy3-nsp13 (bottom). Unwinding reactions were conducted using 10 nM nsp13 (top) or 10 nM Cy3-nsp13 (bottom) in the presence of 5 mM ATP. The unwinding reactions were terminated with quenching buffer after the designated reaction times indicated in the gel images. (**C**) The unwinding fractions of nsp13 (black) and Cy3-nsp13 (green) were quantified over time via PAGE images (B). Each data point represents the mean of three replicates. The error bars denote the SEMs.

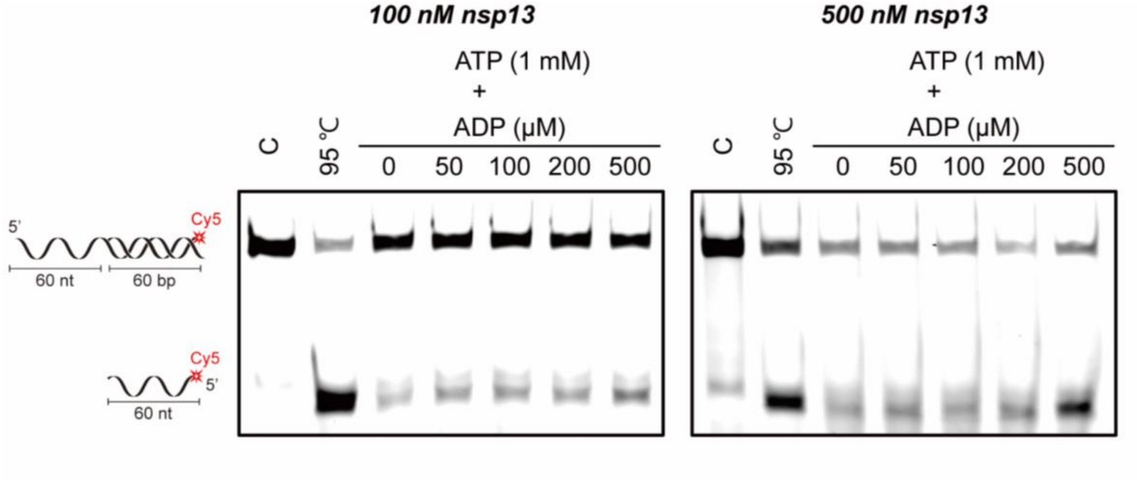

Supplementary Figure S7. Enhanced unwinding activity of nsp13 in the presence of additional ADP.

Representative native PAGE images of the unwinding assay at 100 nM nsp13 (left) and 500 nM nsp13 (right) in the presence of 1 mM ATP but with various additional ADP concentrations (0, 50, 100, 200, and 500 μM). The quantitatively analyzed plot is shown in Figure 6A.

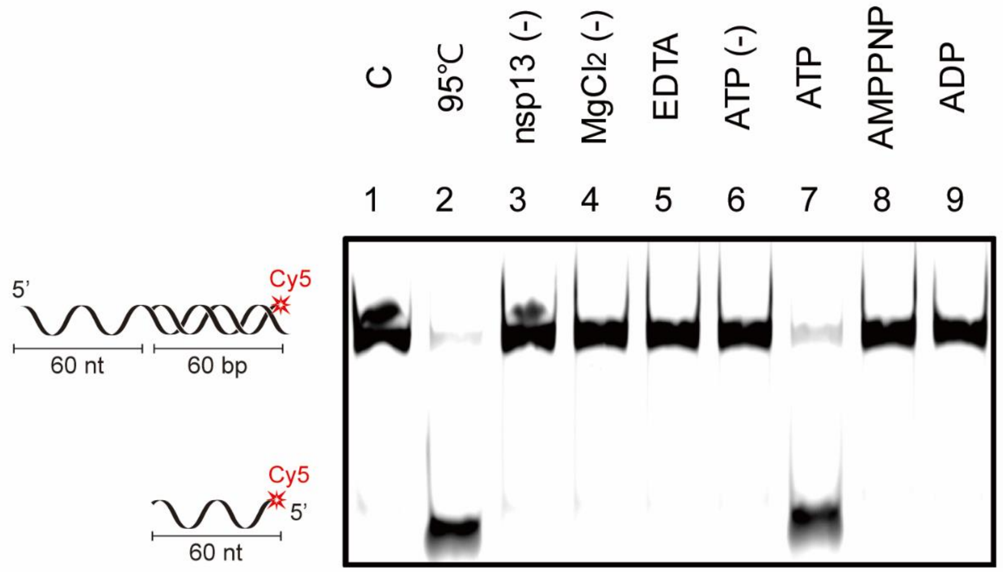

Supplementary Figure S8. In the presence of ADP alone, nsp13 is unable to notably unwind substrates.

A representative native PAGE image of the unwinding reaction at 500 nM nsp13. The unwinding reaction was performed in a reaction mixture containing nsp13, $MgCl_2$, and ATP (see details in the Material and Methods section for the PAGE-based unwinding assay). Lane 1: substrate alone. Lane 2: the product heated at 95 °C for 10 min. Lane 3: a mixture without nsp13. Lane 4: a mixture omitting $MgCl_2$. Lane 5: mixture with EDTA but without $MgCl_2$. Lane 6: a mixture omitting ATP. Lane 7: a mixture with ATP. Lane 8: a mixture with AMPPNP. Lane 9: a mixture with ADP.

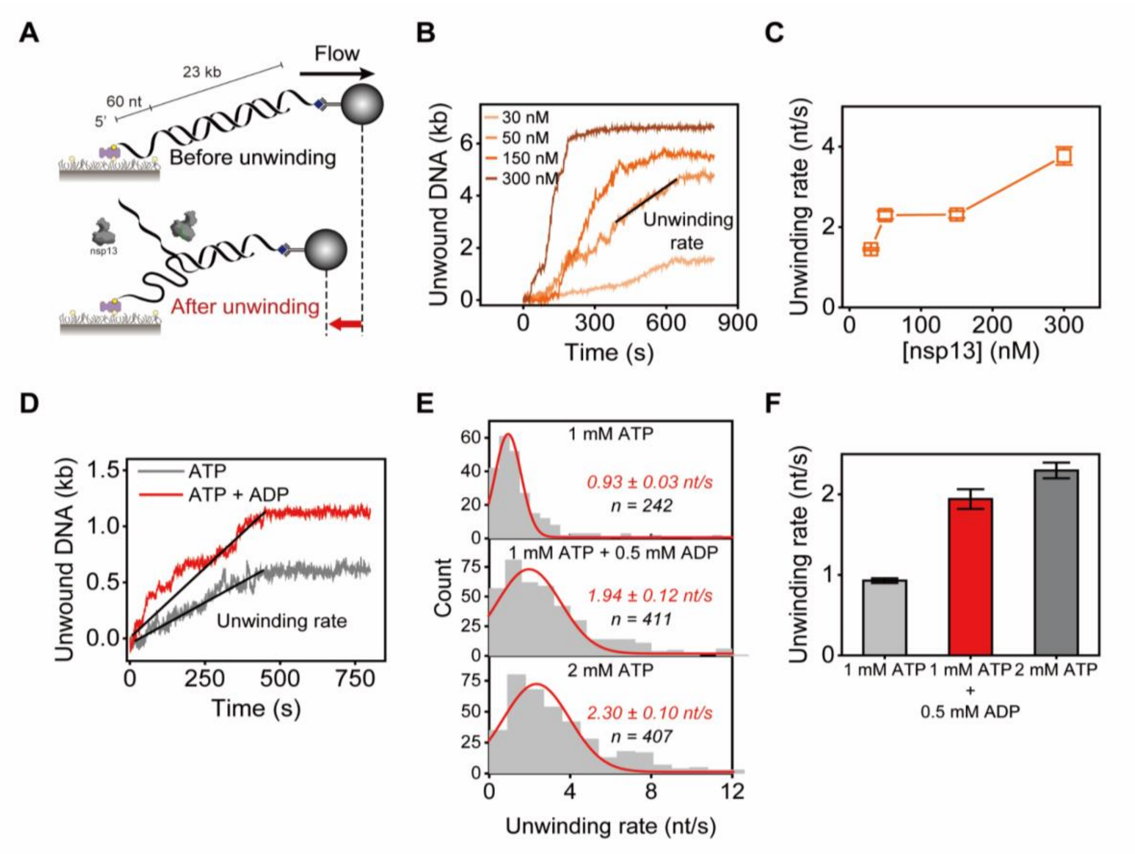

Supplementary Figure S9. The smFS technique shows that unwinding is enhanced by the cooperation of ATP-driven helicase and ADP-dependent chaperone.

(**A**) Schematic of the single-molecule DNA flow-stretching assay. The unwinding activity of nsp13 was measured by tracking the change in the real-time positions of tethered magnetic beads on a modified DNA substrate of ~23 kb. The unwinding activity of nsp13 shortens the bead position by converting dsDNA before the reaction (top) into ssDNA after the reaction (bottom). (**B**) Representative time trajectories at various nsp13 concentrations. The number of nucleotides unwound was determined by the difference in length per base pair between L_{dsDNA} = 0.34 nm and L_{ssDNA} = 0.67 nm. (**C**) Unwinding rate versus nsp13 concentration. The unwinding rate is determined from the slope (as shown by the black line in B). (**D**) Representative time trajectories of unwound DNA in the presence of ATP (gray) or ATP and ADP (red). The black line illustrates how the overall rate is determined by linear fitting. (**E**) Histograms of the unwinding rates of nsp13 at 50 nM in the presence of 1 mM ATP (top), 1 mM ATP and 0.5 mM ADP (middle), or 2 mM ATP (bottom). (**F**) Average unwinding rates measured as in (E). The error bars denote the standard errors.

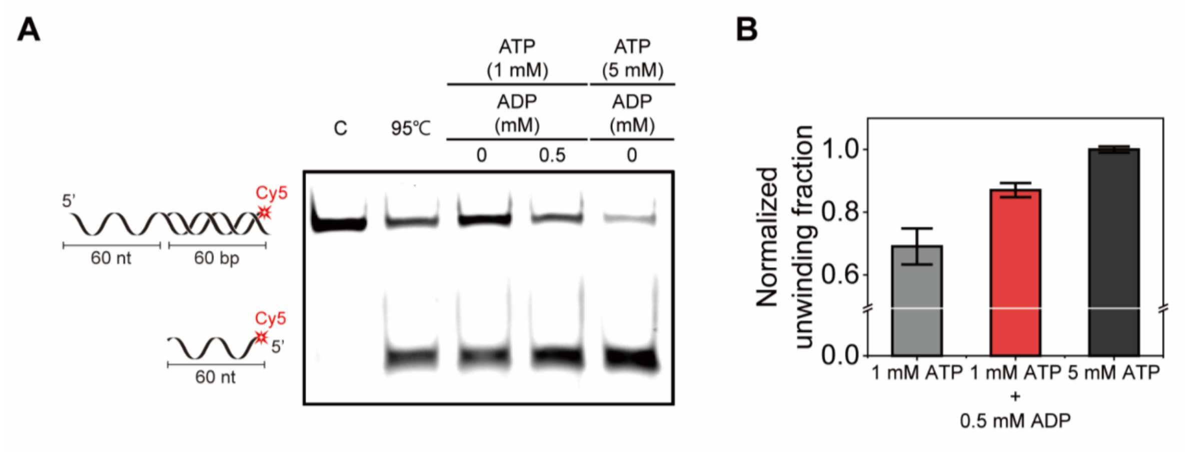

Supplementary Figure S10. The enhancement of nsp13 unwinding by ADP addition was comparable to the increase in saturated unwinding activity at a high ATP concentration of 5 mM.

(**A**) A representative native PAGE gel image showing the unwinding activity of nsp13. Unwinding reaction of nsp13 with (or without) the addition of 0.5 mM ADP at ATP concentrations of 1 mM or 5 mM (indicated at top). The upper and lower bands represent 60 nt-60 bp dsDNA (Supplementary Table S1) and 60 nt ssDNA, respectively, as unwound products. (**B**) Quantification of the nsp13 unwinding products shown in (A) by normalizing the intensities with respect to 5 mM ATP. Compared to that at 5 mM ATP (dark gray), the unwinding fraction at 1 mM ATP is ~ 0.69 (gray), while that at 1 mM ATP with 0.5 mM ADP is ~ 0.87 (red). Each data point represents the mean of three replicates. The error bars denote the SEMs.

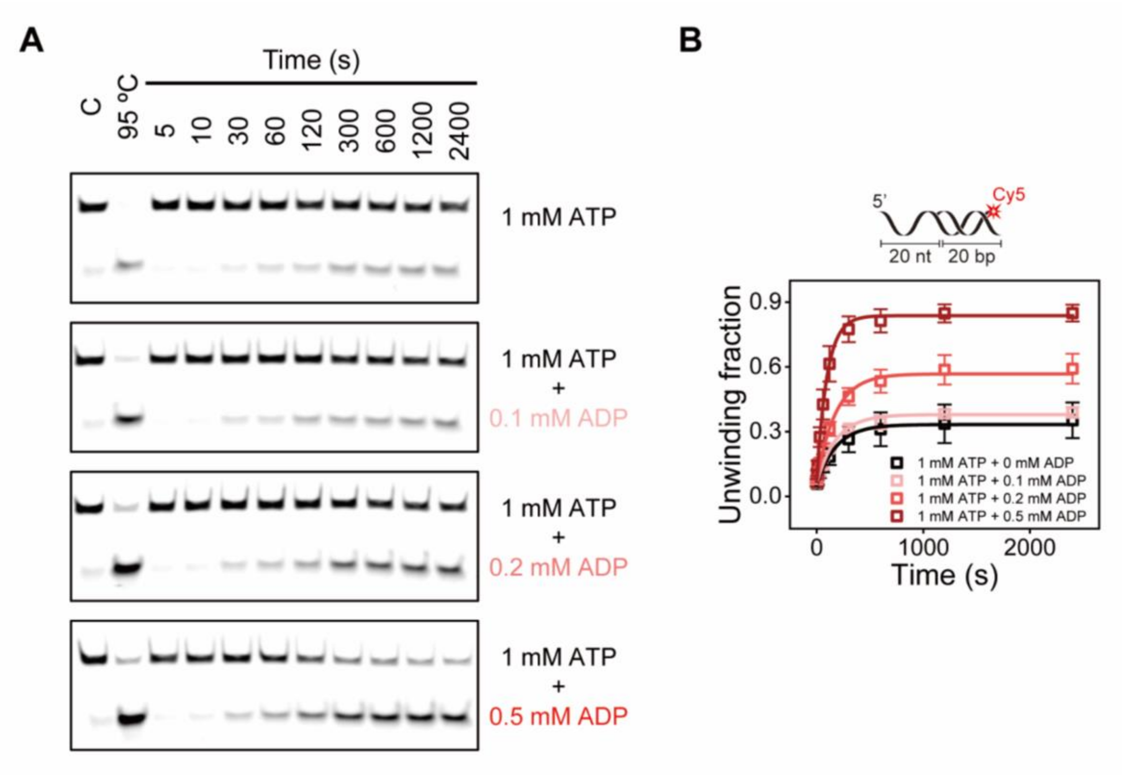

Supplementary Figure S11. ADP addition at a high salt concentration of 100 mM NaCl enhanced the unwinding activity of nsp13.

(**A**) Representative native PAGE images of the unwinding reaction for the 20 nt-20 bp dsDNA (Supplementary Table S1) at 10 nM nsp13, under conditions of 100 mM NaCl, measured at the indicated time points. The reactions were performed in the presence of 1 mM ATP with varying concentrations of ADP (0, 0.1, 0.2, and 0.5 mM) (top to bottom). (**B**) The unwinding fractions of nsp13 were quantified over time via PAGE images (A). Each data point represents the mean of three replicates. The error bars denote the SEMs.

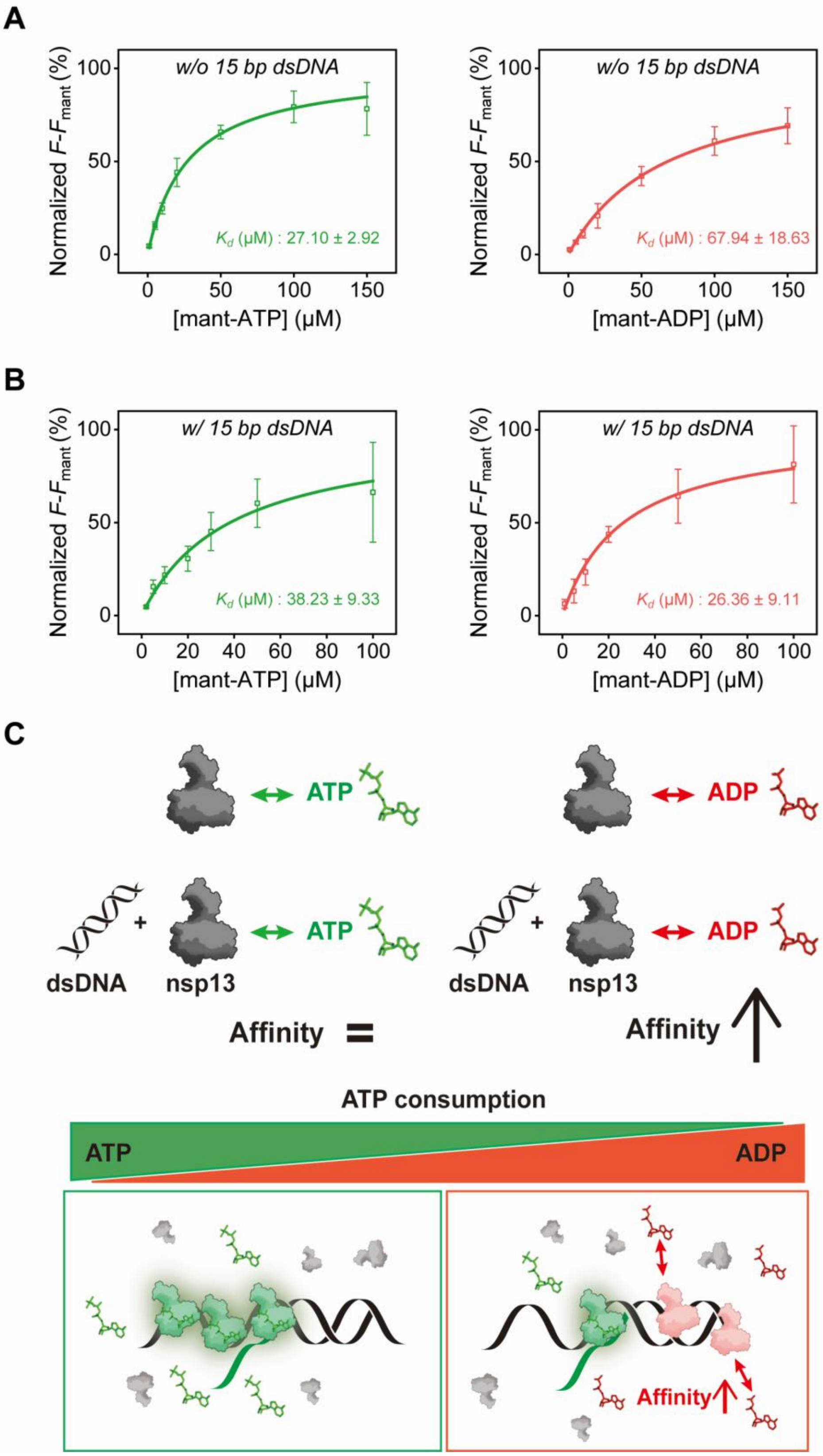

A
w/o 15 bp dsDNA
Normalized F-F mant (%)
[mant-ATP] (μM)
Kd (μM) : 27.10 ± 2.92
[mant-ADP] (μM)
Kd (μM) : 67.94 ± 18.63
B
w/ 15 bp dsDNA
Kd (μM) : 38.23 ± 9.33
Kd (μM) : 26.36 ± 9.11
C
ATP
ADP
dsDNA
nsp13
Affinity
ATP consumption
Affinity

Supplementary Figure S12. Binding affinity of nsp13 to mant-nucleotide.

(**A, B**) The binding affinity of nsp13 to mant-ATP (left) and mant-ADP (right) in the absence (A) or presence (B) of 15 bp dsDNA. The measured fluorescence was normalized by subtracting the intrinsic fluorescence intensity of the mant-nucleotide. Each data point represents the mean of more than three replicates. The error bars denote the SEMs. The K_d values were determined by fitting the data to the Michaelis-Menten model (green or red line). (**C**) Proposed model of nsp13 binding affinities to ATP or ADP in the presence of dsDNA.

Supplementary Table S1. Substrates information

Assay	Cartoon of substrates	Name of substrates	Corresponding figures
**smFRET-based unwinding & Fluctuation**	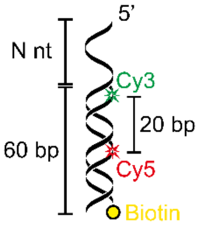	*N* nt-60 bp dsDNA	Figure 1<br>Figure 2<br>Figure 5<br>Supplementary Figure S1<br>Supplementary Figure S3<br>Supplementary Figure S4<br>Supplementary Figure S5
**Binding ability**	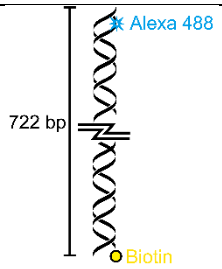	722 bp dsDNA	Figure 3
**DNA T_m shift & Binding affinity**	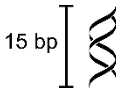	15 bp dsDNA	Figure 4<br>Figure 7C, D<br>Supplementary Figure S12
**PAGE-based unwinding**	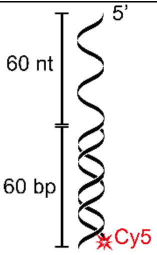	60 nt-60 bp dsDNA	Figure 6A, C<br>Supplementary Figure S2B<br>Supplementary Figure S7<br>Supplementary Figure S8<br>Supplementary Figure S10
	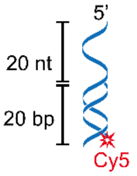	20 nt-20 bp dsRNA	Figure 6B, D
	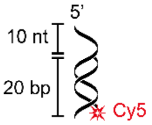	10 nt-20 bp dsDNA	Figure 7A
	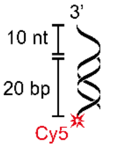	10 nt-20 bp dsDNA (3’OH)	Figure 7B
	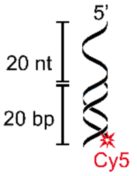	20 nt-20 bp dsDNA	Supplementary Figure S2A<br>Supplementary Figure S6<br>Supplementary Figure S11

**smFS-based unwinding**	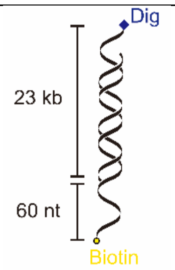	60 nt-23 kb dsDNA	Supplementary Figure S9

Supplementary Table S2. Oligo and primer sequence and modification information

Assay	Name of substrates<br>Sequence name<br>: sequence and modification information	Corresponding figures
	***N* nt-60 bp dsDNA**	
**smFRET-based unwinding & Fluctuation**	Bio_45 nt_Cy5 (45 nt)<br>: Biotin-TAA TAC GAC TCA CTA TAG GGA CAC AAA AAC AAA AT(Cy5)A ACA AGA AAA<br>Phos_15 nt_Cy3 (15 nt)<br>: Phosphate-CAG AAC AAA T(Cy3)AA AAA<br>0 nt tail DNA (60 nt)<br>: TTT TTA TTT GTT CTG TTT TCT TGT TAT TTT GTT TTT GTG TCC CTA TAG TGA GTC GTA TTA<br>15 nt tail DNA (75 nt)<br>: TTT TTT GTG TTT GTT TTT TTA TTT GTT CTG TTT TCT TGT TAT TTT GTT TTT GTG TCC CTA TAG TGA GTC GTA TTA<br>30 nt tail DNA (90 nt)<br>: TTT TTT GTG TTT GTT TTG GTT TAT TTG GTG TTT TTA TTT GTT CTG TTT TCT TGT TAT TTT GTT TTT GTG TCC CTA TAG TGA GTC GTA TTA<br>45 nt tail DNA (105 nt)<br>: TTT TTT TTT TTT TTT TTT TTT GTG TTT GTT TTG GTT TAT TTG GTG TTT TTA TTT GTT CTG TTT TCT TGT TAT TTT GTT TTT GTG TCC CTA TAG TGA GTC GTA TTA<br>60 nt tail DNA (120 nt)<br>: TTT TTT TTT TTT TTT TTT TTT TTT TTT TTT TTT TTT GTG TTT GTT TTG GTT TAT TTG GTG TTT TTA TTT GTT CTG TTT TCT TGT TAT TTT GTT TTT GTG TCC CTA TAG TGA GTC GTA TTA<br>90 nt tail DNA (150 nt)<br>: TTT TTT TTT TTT TTT TTT TTT TTT TTT TTT TTT TTT TTT TTT TTT TTT TTT GTG TTT GTT TTG GTT TAT TTG GTG TTT TTA TTT GTT CTG TTT TCT TGT TAT TTT GTT TTT GTG TCC CTA TAG TGA GTC GTA TTA	**Figure 1**<br>**Figure 2**<br>**Figure 5**<br>**Supplementary Figure S1**<br>**Supplementary Figure S3**<br>**Supplementary Figure S4**<br>**Supplementary Figure S5**
	**60 nt-722 bp dsDNA and 722 bp dsDNA**	
**Binding ability**	Bio_F_primer (23 nt)<br>: Biotin-AAA AAG GTG AGC CGG TCA CCT GG<br>Phos_15 nt_R_primer (22 nt)<br>: Phosphate-CCT CCG GCG TGC TTA CCA CGA A<br>Phos_linker DNA (59 nt)<br>: Phosphate-AAG CAC GCC GGA GGT AAT ACG ACT CAC TAT	**Figure 3**

	AGG GAC ACA AAA ACA AAA TAA CAA GAA AA<br>Phos 15 nt Alexa488 (15 nt)<br>: Phosphate-CAG AAC AAA T(Alexa 488)AA AAA<br>Blunt tail DNA (60 nt)<br>: TTT TTA TTT GTT CTG TTT TCT TGT TAT TTT GTT TTT GTG<br>TCC CTA TAG TGA GTC GTA TTA	
**DNA**<br>**T_m shift**<br>**&**<br>**Binding affinity**	**15 bp dsDNA**<br>15 ds F (15 nt)<br>: TCT CAC TAA ATC CAA<br>15 ds R (15 nt)<br>: TTG GAT TTA GTG AGA	**Figure 4**<br>**Figure 7C, D**<br>**Supplementary Figure S12**
	**60 nt-60 bp dsDNA and trap DNA**<br>Gel 60 nt Cy5 DNA (60 nt)<br>: Cy5-TAA TAC GAC TCA CTA TAG GGA CAC AAA AAC AAA<br>ATA ACA AGA AAA CAG AAC AAA TAA AAA<br>60 nt tail DNA (120 nt)<br>: TTT TTT TTT TTT TTT TTT TTT TTT TTT TTT TTT TTT GTG TTT<br>GTT TTG GTT TAT TTG GTG TTT TTA TTT GTT CTG TTT TCT<br>TGT TAT TTT GTT TTT GTG TCC CTA TAG TGA GTC GTA TTA<br>60 nt trap DNA (60 nt)<br>: TAA TAC GAC TCA CTA TAG GGA CAC AAA AAC AAA ATA<br>ACA AGA AAA CAG AAC AAA TAA AAA	**Figure 6A, C**<br>**Supplementary Figure S2B**<br>**Supplementary Figure S7**<br>**Supplementary Figure S8**<br>**Supplementary Figure S10**
**PAGE-based**<br>**unwinding**	**20 nt-20 bp dsRNA and trap RNA**<br>Gel 20 nt Cy5 RNA (20 nt)<br>: Cy5-rGrGrU rGrGrU rGrGrU rArArG rArUrG rArUrG rArG<br>Gel 40 nt RNA (40 nt)<br>: rUrUrU rUrUrU rUrUrU rUrUrU rUrUrU rUrUrU rUrUrC<br>rUrCrA rUrCrA rUrCrU rUrArC rCrArC rCrArC rC<br>20 nt trap RNA (20 nt)<br>: rGrGrU rGrGrU rGrGrU rArArG rArUrG rArUrG rArG	**Figure 6B, D**
	**10 nt-20 bp dsDNA and trap DNA**<br>Gel 20 nt Cy5 DNA (20 nt)<br>: Cy5-GGT GGT GGT AAG ATG ATG AG<br>10 nt tail DNA (30 nt)<br>: TTT TTT TTT TCT CAT CAT CTT ACC ACC ACC<br>20 nt trap DNA (20 nt)<br>: GGT GGT GGT AAG ATG ATG AG	**Figure 7A**
	**10 nt-20 bp dsDNA (3′OH) and trap DNA**<br>Gel 30 nt Cy5 DNA (30 nt)<br>: Cy5-GGT GGT GGT AAG ATG ATG AGA AAA AAA AAA	**Figure 7B**

	20 nt complementary DNA (20 nt)<br>: CTC ATC ATC TTA CCA CCA CC<br>30 nt trap DNA (30 nt)<br>: GGT GGT GGT AAG ATG ATG AGA AAA AAA AAA	
	**20 nt-20 bp dsDNA and trap DNA**<br>Gel 20 nt Cy5 DNA (20 nt)<br>: Cy5-GGT GGT GGT AAG ATG ATG AG<br>Gel 40 nt DNA (40 nt)<br>: TTT TTT TTT TTT TTT TTT TTC TCA TCA TCT TAC CAC CAC C<br>20 nt trap DNA (20 nt)<br>: GGT GGT GGT AAG ATG ATG	**Supplementary Figure S2A**<br>**Supplementary Figure S6**<br>**Supplementary Figure S11**
**smFS-based unwinding**	**60 nt-23 kb dsDNA**<br>Bio_60T_18 nt DNA (78 nt)<br>: Biotin-TTT TTT TTT TTT TTT TTT TTT TTT TTT TTT TTT TTT TTT TTT TTT TTT TTT TTT TTT TTT GAA CGA TCA GCA AAG TAA<br>Phos_linker DNA (30 nt)<br>: Phosphate-AGG TCG CCG CCC TTA CTT TGC TGA TCG TTC<br>Phos_7 nt_Dig (7 nt)<br>: Phosphate-AGC TTT T-Digoxigenin	**Supplementary Figure S9**
